# Environmental Drivers on Blue Tit Nest Microbiome: An Experimental Study

**DOI:** 10.1002/ece3.74007

**Published:** 2026-07-14

**Authors:** Marina García‐del Río, Tamara Martin‐Pozas, Sergio Sanchez‐Moral, Alejandro Cantarero, Francisco Castaño‐Vázquez, Yago Merino, Javier García‐Velasco, Santiago Merino

**Affiliations:** ^1^ Department of Evolutionary Ecology National Museum of Natural Sciences, Spanish National Research Council (CSIC) Madrid Spain; ^2^ Institute of Natural Resources and Agrobiology of Seville (IRNAS) Spanish National Research Council (CSIC) Seville Spain; ^3^ Department of Geology National Museum of Natural Sciences, Spanish National Research Council (CSIC) Madrid Spain; ^4^ Department of Physiology, Veterinary School Complutense University of Madrid Madrid Spain

**Keywords:** bacteria, fungi, humidity, microclimate, temperature

## Abstract

Microclimate inside avian nesting cavities provides suitable growth conditions for microbial communities, which in turn may play a crucial role in influencing the well‐being of the host. In this study, we investigated the microbiome of Blue Tit ( *Cyanistes caeruleus* ) nests subjected to experimental manipulations of temperature and humidity, aiming to evaluate the impact of these factors on fungal and bacterial communities. Additionally, we examined the associations between these microbial communities, parasitism and nesting birds' condition. Our results, based on metabarcoding analysis using 16S rRNA and the ITS2 region, indicated that while bacterial alpha diversity remained unaffected by the experimental manipulation, beta diversity differed significantly, particularly between nests with increased humidity and control nests. Similarly, elevated temperature and humidity increased fungal richness (alpha diversity) and altered fungal composition (beta diversity). We also observed that the abundances of bacterial and fungal phyla varied between treatments, with the differences being most pronounced in the case of fungi. We did not detect significant differences in potentially pathogenic bacteria between treatments. However, potentially pathogenic fungi, including dermatophytes, proliferated in humidified nests, potentially contributing to poorer nestling body condition compared to other nest treatment groups. The study also revealed significant correlations between microbial communities, ectoparasites and nestling body condition, indicating their potential interconnection. To our knowledge, this study represents the first experimental analysis of the microclimate effects on the nest microbiome. These findings highlight the complex interactions between nest microclimate, microbial diversity, ectoparasites and nestling development, offering new insights into the ecological effects of microclimatic conditions in avian nesting environments.

## Graphical abstract

Elevated humidity and temperature altered nest microbial composition and its associations with parasitism and nestling condition. These findings identify nest microclimate as an important driver of nest microbiomes with consequences for avian development.

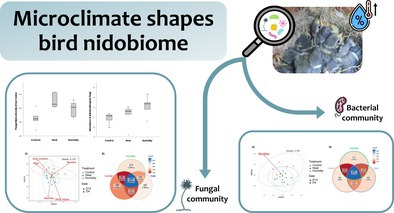

## 1 Introduction

The relationships between microorganisms and birds constitute complex associations, encompassing various beneficial functions for the host, including nutrition, defence and protection (Soler et al. 2010). In particular, the microbiota can inhibit pathogen colonisation and promote host growth and development (Audisio et al. 2000; Moreno et al. 2003). However, some microbes are avian pathogens, either obligate (i.e., requiring a host to complete their life cycle) or facultative (i.e., typically free‐living but able to adopt a parasitic lifestyle when encountering suitable conditions or a susceptible host), and can have harmful effects on the host's health such as tissue damage, impaired immune function, reduced reproductive success, and, in severe cases, mortality (Fiennes 1982; Nuttall 1997; Malik et al. 2021; Costanzo et al. 2022). For example, numerous bacteria residing in avian plumage can affect plumage condition and colouring (Burtt Jr and Ichida 1999, 2004; Shawkey et al. 2007).

In the case of nesting animals, the nest microbiome influences both the well‐being of the host and its offspring, with microbial communities transferring among nest materials, adult birds and chicks, and collectively influencing avian reproductive development (Pugh 1996). Recent studies have demonstrated that the gut microbiota differs between nestlings and adults and undergoes changes across developmental stages, highlighting a dynamic process of microbiome maturation (Zhang et al. 2025). However, few studies have been conducted on the microbial species associated with the nesting environment of free‐living passerines (Mills et al. 1999; Berger et al. 2003; González‐Braojos, Vela, Ruiz‐De‐Castañeda, Briones, Cantarero, and Moreno 2012; van Veelen et al. 2018; Chen et al. 2020; Campos‐Cerda et al. 2023; Diez‐Méndez et al. 2023; Yang et al. 2025), and consequently, the understanding of variation in nest bacterial and fungal assemblages in this environment is limited (Hubálek 1978; Singleton and Harper 1998). Indeed, associations between wild birds and fungi are rarely reported, and the few studies published have typically focused on bacteria (Cafarchia et al. 2006; Yang et al. 2025).

The nest microbiome can interact with its hosts in many different ways, including mutual transfer of microbes between birds and nest materials, which plays an important role in the nestling growth and reproduction of birds (Pugh 1996). In fact, breeding birds are at an increased risk of bacterial and fungal infections (González‐Braojos, Vela, Ruiz‐De‐Castañeda, Briones, Cantarero, and Moreno 2012). For example, birds are particularly susceptible to infections of the lungs and air sacs by *Aspergillus fumigatus* , a fungus isolated from nests (Barathidasan et al. 2013). A variety of potentially pathogenic microbiota were also isolated from nests of different species of birds, which may affect nestling development (David et al. 1998; Barathidasan et al. 2013; Korniłłowicz and Kitowski 2013). In addition, the bacterial communities on the eggshell may negatively affect hatching success (Peralta‐Sánchez et al. 2012) but may also contribute to host protection by promoting antibiotic‐producing taxa that inhibit potential pathogens (Song et al. 2023). Moreover, the role of the nest environment in shaping the gut microbiome of birds is complex and results from the interaction of multiple factors, including host genetics, environmental conditions and host behavioural responses (Lucas and Heeb 2005; Teyssier et al. 2018; Costanzo et al. 2022). This complexity is captured by the concept of the ‘nidobiome’, a new unit of microbiome–host interactions (Campos‐Cerda and Bohannan 2020), which proposes that the quantitative contributions of host genetic variation, environmental variation (including nest architecture) and variation in plastic host responses (e.g., learned nesting behaviour) interact with each other to determine the neonate's microbiome and its fitness consequences. For instance, cavity nests provide optimal conditions for bacterial growth due to their stable microclimatic environment (Mehmke et al. 1992; Singleton and Harper 1998; Goyache et al. 2003; González‐Braojos, Vela, Ruiz‐De‐Castañeda, Briones, Cantarero, and Moreno 2012; Costanzo et al. 2022). Furthermore, cavity‐nesting birds such as *Parus monticolus* have been shown to actively modify the nest microecology during the post‐construction stage, thereby contributing to the establishment of a stable and health‐promoting microenvironment for offspring development (Yang et al. 2025).

Additionally, ectoparasites may influence the diversity and composition of microbial communities in bird nests (Tomás et al. 2018), while microbial metabolites can simultaneously affect parasite behaviour. For instance, volatile compounds produced during bacterial metabolism are used by some ectoparasites as olfactory cues for host detection (Mazorra‐Alonso et al. 2024), while host selection within nests is additionally shaped by host intrinsic and behavioural traits, with blood‐feeding insect vectors preferentially biting nestlings according to their sex, body mass and position within the nest (García‐del Río et al. 2024), together ultimately modulating parasite infestation intensity and fledging success.

Nest‐dwelling ectoparasites, including fleas, larval dipterans, mites and ticks, feed on the blood of both adult and nestling birds (Loye and Zuk 1991; Merino 2010; López‐Rull and Macías‐Garcia 2015). Their feeding activity generates organic residues, such as damaged skin, blood remains and parasite faeces, which accumulate within nests and may provide substrates that facilitate bacterial colonisation and growth (Weiss 2006), including that of potentially pathogenic species. These findings suggest that ectoparasites may act as important mediators of indirect interactions within nest environments, although this possibility has been very little explored (but see Tomás et al. 2018).

Climatic conditions may further modulate these interactions by altering the stability and composition of host‐associated microbial communities (González‐Braojos, Vela, Ruiz‐de‐Castañeda, Briones, and Moreno 2012). However, relatively few studies have investigated how temperature and humidity jointly affect the avian microbiome and its interactions with ectoparasites. Most field‐based studies relate ambient temperature to microbiome variations across seasons or geographical locations, but often overlook other environmentally relevant factors, such as humidity and rainfall (Campbell et al. 2020; Gaona et al. 2020). Likewise, field‐based microbiome research seldom examines the interaction between temperature, humidity and other environmental stressors, including parasitism (Ingala et al. 2021).

The relationship between avian microbial communities and birds is pivotal in shaping the evolution of their life‐history traits (Soler et al. 2015). Nevertheless, studies examining bird‐microbiome interactions have predominantly focused on gut microbiota, while the microbial ecology within nests remains significantly understudied. All this highlights a limited understanding of the interactions occurring within the nest, despite existing studies providing evidence of links between microbial activity and parasites mediated by the gases produced in the nests (Castaño‐Vázquez et al. 2020, 2025) that underscore the complexity of such relationships.

For these reasons, in this study we analysed the microbiome of bird nests where temperature and humidity were experimentally manipulated to evaluate the impact of these factors on fungal and bacterial communities, as well as their associations with parasitism and the body condition of the birds. We anticipate that bacterial and fungal communities will change in response to modifications in the nest microclimate. Specifically, we expect an increase in potentially harmful parasites, as higher temperatures and humidity may provide optimal conditions for their proliferation. These altered communities could partially replace the original ones, disrupting the natural balance and, in turn, affecting their interactions with both the birds and the parasites inhabiting the nest. Furthermore, we expect that manipulation promotes potentially microbial communities detrimental to nestling growth, given that nestlings in nests with altered microclimatic conditions exhibited poorer development (García‐del Río et al. 2025).

## 2 Methods

### 2.1 Study Population

This study was carried out during the 2021 bird breeding season in a montane deciduous forest of Pyrenean oak *Quercus pyrenaica* located in Valsaín (Segovia, central Spain, 40°53′74″ N, 4°01′W, 1200 m a.s.l.). A Blue Tit ( *Cyanistes caeruleus* ) population breeding in wooden nest boxes hanging from tree branches about 4 m above the ground has been studied in this area since 1991 (Sanz 2002; Tomas et al. 2006). Each breeding season, nest boxes are emptied at the beginning of the breeding season and periodically inspected to determine reproductive parameters, including laying date, clutch size, hatching date and brood size at fledging (Merino et al. 2000; Tomas et al. 2007).

The Blue Tit is a cavity‐nesting bird widely distributed across the western Palearctic (Cramp and Perrins 1993) that readily uses nest boxes for breeding. Most Blue Tit populations are sedentary, as is the case in our study population. Blue Tits during the breeding season are insectivorous and females can be easily differentiated from males by the presence of a brood patch on the abdomen. Also, they present a slightly sexually dichromatic plumage, with males being more intensely coloured than females (Cramp and Perrins 1998). Females lay a single clutch with an average of 9.1 eggs and produce an average of 7.8 nestlings in this population (Fargallo and Johnston 1997).

Blue Tit nests are characterised by the presence of biting flying insects and nest‐dwelling ectoparasites such as blowflies (*Protocalliphora azurea*), a dipteran flying insect whose larvae feed on blood and severe infestations may be lethal for nestlings (Whitworth and Bennett 1992; Merino and Potti 1995, 1996; Hurtrez‐Bousses et al. 1997; Cantarero et al. 2013); fleas (*Ceratophyllus gallinae*), where only the adults feed on blood and are able to adversely affect the growth and survival of nestlings (Richner et al. 1993); haematophagous mites (*Dermanyssus* spp.), which can act as vectors for trypanosomes (Macfie and Thomson 1929) and can cause anaemia and affect the growth of nestlings (Merino and Potti 1995, 1996); biting midges (*Culicoides* spp.), which are very small dipterans, known to be vectors of haemosporidian parasites of the genus *Haemoproteus* (Valkiūnas 2005; Martínez‐de la Puente et al. 2011), and to have detrimental effects on bird reproduction and survival (Merino et al. 2000; Martínez‐de la Puente et al. 2010); and blackflies (Diptera: Simuliidae), which are vectors of another common blood parasite of birds, *Leucocytozoon* spp. (Order Haemosporida) (Merino et al. 2000; Martínez‐de la Puente et al. 2010) and have an impact on the health of adults and nestlings, causing dermatitis and anaemia (Hunter et al. 1997; Smith et al. 1998; Bukacijski and Bukacijska 2000).

### 2.2 Study Design

During the 2021 bird breeding season (from April to June), an experiment was carried out on the effects of experimental alteration of temperature and humidity on nest‐dwelling ectoparasites and their bird hosts (see García‐del Río et al. 2025 for the published data). The nests were grouped in triads according to hatching date and brood size (±1 day and ±1 nestling) and each nest box was randomly assigned to one of three experimental categories: heat treatment, humidity treatment, or control group. In total, 47 nest boxes (16 heated, 15 humidified and 16 control) were used in the experiment. A sub‐sample of nests from the experiment described above was selected for microbiome analysis of the nest material (see below). Experimental nests were equipped with heat mats (70 × 70 mm, 5 V/3.5 W; thermo Flächenheizungs GmbH, Germany) placed on the floor of the nest box and separated from nest material by a metal grid that provided heat 24 h a day. In addition, sponges moistened with water and moisture‐preserving gel (Aquaplant Complet Gel—FLOWER composed of water, microcellulose and monopotassium phosphate) were placed above the metal grid in contact with the underside of the nest material in nests with humidity treatment. Metal grids and cords were also installed in control nests during the experiment, which were visited as frequently as the heated and humidified nests. All the nest boxes were also fitted with sensors that register both temperature and relative humidity during the experimental period (Thermochron DS1923; 6 × 17 mm, temperature range: −20 to 85°C; resolution 0.0625°C; humidity range: 0%–100% with a resolution of 0.04%; Maxim IC, USA). The experiment lasted 14 days in each nest box, from Day 3 to 17 post‐hatching. Once nestlings fledged (Day 20 post‐hatching), sensors were removed and the nest material was collected for ectoparasite quantification. See Table 1 for a summary of the parasites and the collection methods used and García‐del Río et al. 2025 for more details.

**TABLE 1:** Species of parasites in Blue Tit nests and the collection methods adopted in this study.

Species of parasites	Collection methods	References
Blowflies (*Protocalliphora azurea*)	Dismantling the nest material	Merino and Potti (1995)
Mites (*Dermanyssus* spp.)	Tullgren funnels	Merino and Potti (1995)
Fleas (*Ceratophyllus gallinae*)	Tullgren funnels	Merino and Potti (1995)
Biting midges (*Culicoides* spp.)	Gel‐plate trap	Tomas et al. (2008)
Blackflies (Diptera: Simuliidae)	Gel‐plate trap	Tomas et al. (2008)

All adult birds were captured in their nest boxes while feeding 14‐day‐old nestlings during the daytime, using a conventional nestbox trap set at the entrance. The trap was placed for a maximum of 1 h and removed once both adults were captured. All birds were supplied with individually numbered rings for identification. The body mass of nestlings aged 14 days was recorded with an electronic balance to the nearest 0.1 g. Tarsus length was measured with a digital calliper to the nearest 0.1 mm. A body condition index was calculated from these two variables as the residuals of a linear regression of body mass on tarsus length.

The full experiment (*N* = 47) resulted in a significant increase in relative humidity in humidified nests (76.15% ± 2.76%) compared to controls (60.45% ± 1.89%) and heated nests (58.63% ± 1.53%), and a significant increase in temperature in heated nests (21.34°C ± 0.46°C) compared to control nests (19.48°C ± 0.31°C). However, there were no significant differences regarding humidified nests (20.33°C ± 0.54°C). See Figure 2 in García‐del Río et al. (2025) for further information on the variation of these factors across treatments. During the period of the experiment, the average temperature in the forest was 14.33°C ± 0.73°C, while the average relative humidity was 62.1% ± 1.96%. This experimental modification significantly affected the abundance of ectoparasites; specifically, there was a lower abundance of blowflies in heated and humidified nests compared to control nests, and a lower abundance of mites in humidified nests compared to control and heated nests. In addition, this experimental modification negatively affected the body condition of the nestlings. We found that the body condition of the nestlings was lower in humidified nests and slightly lower in heated nests, as compared to control nests (see García‐del Río et al. 2025 for more details).

### 2.3 Microbiome Sampling

We randomly selected three nest triads, so a total of nine nests were sampled (3 heated, 3 humidified and 3 control). Each nest was sampled on two different days of nestling development, Day 4 and Day 13. Therefore, a total of 18 samples belonging to 9 different nests were collected. Samples from the nest cup, where nestlings were placed in the nest, were taken using sterile cotton‐tipped swabs. First, we put on nitrile gloves sterilised with hydroalcoholic gel to extract nestlings from the nests. Then the nest cup was swabbed for 30 s by the same person in a uniform pattern with a particular effort to keep this process standardised, avoiding inter‐operational variability. The person who collected the samples did not wear a mask but maintained their head as far as possible from the nest and sampling swabs to avoid contamination. Sterile swabs were used to collect the samples, which were then placed into sterile 2 mL cryotubes and covered in DNA/RNA Shield for DNA preservation and nestlings were returned to the nest. Samples were transported to the lab within 24 h and stored at −80°C until the DNA extractions were performed.

### 2.4 DNA Extraction and Sequencing

DNA extraction and library preparation were conducted by AllGenetics & Biology SL (https://www.allgenetics.eu/) using NovaSeq PE250 (Illumina) for generating 250 bp paired‐end reads. DNA was extracted using the Zymo Microprep (ZymoBIOMICS), following the manufacturer's instructions. Extractions and PCR blanks were included to control for possible contamination. The primers Bakt_341F (5′ CCTACGGGNGGCWGCAG 3′) and Bakt_805R (5′ GACTACHVGGGTATCTAATCC 3′) were used to amplify the V3‐V4 regions of the 16S rRNA gene for bacterial library preparation (Herlemann et al. 2011). For fungal library preparation, the primers ITS86F (5′ GTGAATCATCGAATCTTTGAA 3′) and ITS4 (5′ TCCCCGCTTATTGATATGC 3′) were used to amplify the ITS2 region (White et al. 1990; Turenne et al. 1999).

### 2.5 Data Analyses

The QIIME2 pipeline was used to process sequencing reads, using DADA2 to remove primers, filter chimeric sequences and infer ASVs from demultiplexed data (Callahan et al. 2016; Bolyen et al. 2019). Taxonomic assignment of ASVs was generated using QIIME2 Naive Bayes classifiers trained with 16S sequences from the SILVA release 138 database for bacteria and ITS fungal sequences from UNITE 9 (Abarenkov et al. 2010; Quast et al. 2013). Amplicon sequence variants (ASVs) detected in negative controls were present at very low abundance and were removed prior to downstream analyses (Table S1).

Data processed through QIIME2 were imported into RStudio with the ‘Phyloseq’ package. ASVs unassigned at the phylum level, as well as those assigned to mitochondria and chloroplasts, were excluded prior to downstream analyses in R. The abundance results of the ASVs expressed in relative abundances at the phylum and genus level were visualised using the ‘ggplot2’ package.

Prior to alpha diversity analyses, bacterial data were rarefied to 44,925 sequences per sample and fungal data to 57,652 sequences per sample, respectively, corresponding to the lowest sequencing depth in each dataset. The rarefied data were used to infer alpha diversity by Shannon's, Simpson's and Chao1 diversity indexes for each sample using the vegan package. The Shannon index accounts for both species' richness and evenness, with higher values indicating greater diversity. The Simpson index reflects species dominance, representing the probability that two randomly selected individuals belong to the same species, where higher values correspond to higher diversity (Simpson index 1–D, where D is the diversity index). The Chao1 index provides an estimate of total species richness, placing particular emphasis on rare or undetected species to correct for sampling limitations. We tested the effects of treatments, as well as the effects of different days of nestling development, on Shannon's, Simpson's and Chao1 diversity indexes. In these models, only treatment and sampling date were included as independent variables. Normality of alpha diversity data was checked using Shapiro–Wilk tests. Parametric tests (ANOVA and *t*‐test) were applied to normally distributed variables, whereas non‐parametric tests (Kruskal–Wallis and Wilcoxon) were used for non‐normal distributions. Briefly, bacterial Shannon and Simpson indices were analysed using parametric tests, whereas Chao1 was analysed using non‐parametric tests. In contrast, for fungi, the Chao1 index was analysed using parametric tests, while Shannon and Simpson indices were analysed using non‐parametric tests. A Venn diagram was used to identify shared and unique ASVs among experimental groups. We used the ‘vegan’ package to assess the beta diversity of nest samples through non‐metric multidimensional scaling (NMDS) ordination, PERMANOVA and ANOSIM analysis, with 999 permutations, using relative abundance data and Bray Curtis distance. Interaction effects between treatment and sampling date were tested in beta diversity analyses (PERMANOVA) but were not significant. Additionally, to identify the parasite abundance (mites, flea larvae, blowflies, black flies and biting midges) and nestling body condition variables that significantly fit with the NMDS ordination, we used the envfit function. To assess the strength and statistical significance of differential abundance of bacterial and fungal taxa across different treatments we used the ‘DESeq2’ package. Finally, to evaluate significant relationships between different parasites, bird condition and microbial communities, we employed the Mantel Test and multiple correlation analysis. Mantel tests were performed to assess the relationship between microbial community dissimilarity (Bray–Curtis distance) and environmental variables using Spearman correlation with 999 permutations. Multiple correlation analyses were assessed using Spearman correlation to compare relative abundances and ecological variables.

FUNGuild was used to predict fungal community ecological roles and functions of fungi within nests (Nguyen et al. 2016). Similarly, FAPROTAX was used to map bacterial clades and predict ecologically relevant functions based on the established functions of cultured strains from current literature (Louca et al. 2016). These analyses were performed to assess the potential pathogenic role of bacterial and fungal communities across treatments.

To explore the relationship between the abundance of bacterial and fungal potential animal pathogens and the experimental treatment, based on FAPROTAX and FUNGuild, respectively, we used generalised mixed models (GLMM) with a normal distribution and identity link function. The abundance of potentially pathogenic bacteria and fungi was used as dependent variables in two different models, the experiment (the three different treatments) and the date (Days 4 and 13) were included as fixed factors, and the nest was included as a random factor. We structured the data as repeated measures, specifying that each nest had two measurements. In addition, pairwise contrasts of estimated means were used as post hoc tests in GLMM analyses. These models were performed in SPSS (IBM Corp. Released 2022. IBM SPSS Statistics for Windows, Version 29.0.0.0 (241) Armonk, NY: IBM Corp). The remaining analyses were performed in R version 4.3.1 (R Core Team 2023) using RStudio (RStudio Team 2023). We used the packages phyloseq (McMurdie and Holmes 2013), vegan (Oksanen et al. 2022), ggplot2 (Wickham 2016) and DESeq2 (Love et al. 2014).

### 2.6 Ethical Note

This study complies with current European legislation on experimental procedures with animals (2010/63/UE) and was reviewed and approved by the Dirección General de Agricultura, Ganaderia y Alimentación, Comunidad de Madrid (Spain), Permission PROEX 128/19. Annual ringing permissions were provided by Junta de Castilla y León.

## 3 Results

### 3.1 Diversity and Abundance of Bacteria

#### 3.1.1 Alpha Diversity

Bacterial alpha diversity measurements did not differ between treatments (Shannon index: *F* = 0.479, *p* = 0.629; Simpson index: *F* = 1.421, *p* = 0.272; Chao1 index: *H* = 0.292, *p* = 0.864) and neither between the sampling dates (Shannon index: *t* = 0.057, *p* = 0.955; Simpson index: *t* = 0.566, *p* = 0.578; Chao1 index: *W* = 36, *p* = 0.730). See Table S2 for more information on the alpha diversity indices in the different treatments.

#### 3.1.2 Beta Diversity

**FIGURE 1:**
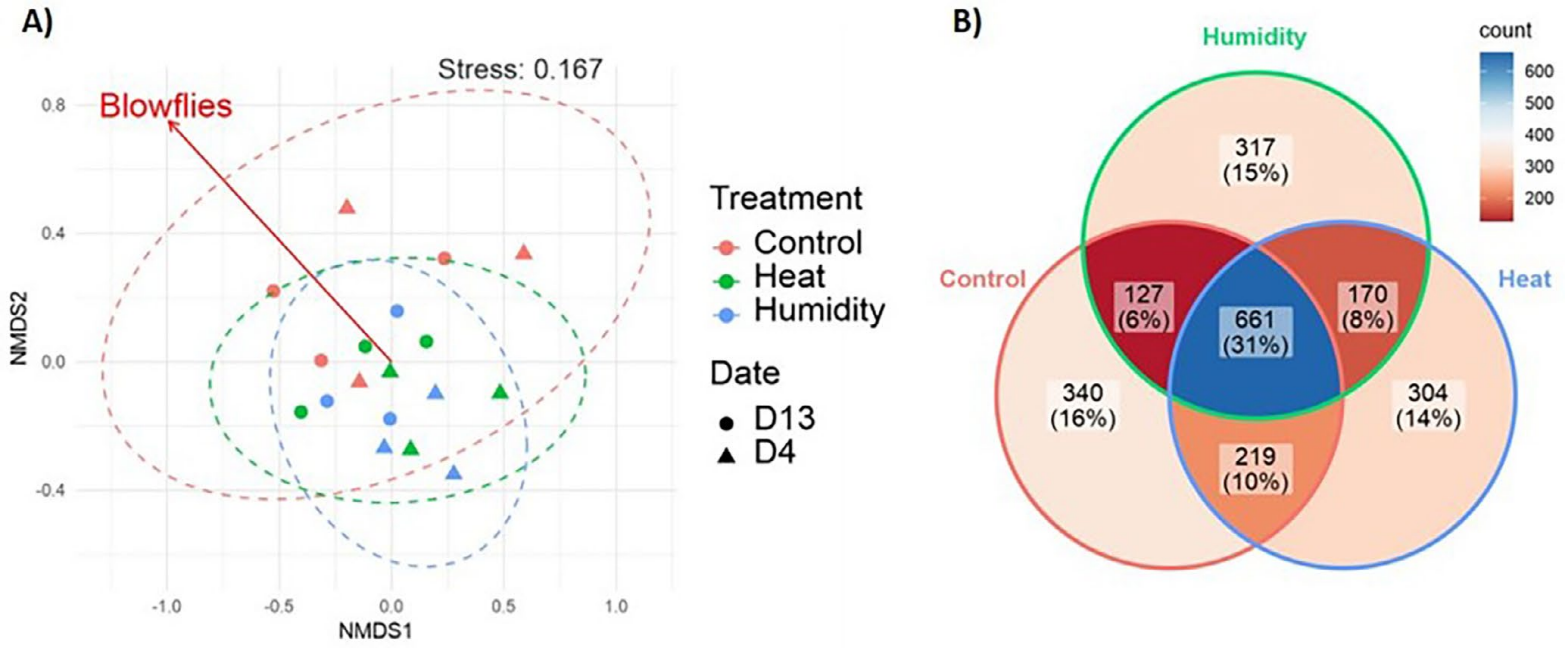
Similarity of the bacterial community (Beta diversity). (A) Non‐metric multidimensional scaling (NMDS) ordination plot. The points are individual samples positioned based on their similarity in bacterial community composition. The ellipses represent the groupings or clusters based on the variable ‘Treatment’ and show the area where 95% of the points within a group are expected to lie. The red arrow indicates the only variable fitted to the NMDS ordination using envfit. The arrow points in the direction of increasing values for that variable. (B) Venn Diagram showing differences or similarities in ASVs at the species level. Each set represents a treatment and the overlap between sets shows which ASVs are shared between them.

We found significant differences between treatments in terms of the similarity of the bacterial community, specifically between control and humidified nests, and a trend between the control and heated nests. However, there were no significant differences between humidity and heated nests and between sampling dates (Table 2; Figure 1).

**TABLE 2:** Differences in the bacterial community (beta diversity) between treatments and sampling date according to ANOSIM and PERMANOVA analyses.

ANOSIM	Sample size	Permutations	*R*	*p*
Date	18	999	0.044	0.221
Treatment	18	999	0.156	**0.045**

Pairwise	Sample size	Permutations	*R*	*p*
Control—Humidity	12	999	0.266	**0.040**
Control—Heat	12	999	0.224	0.074
Humidity—Heat	12	999	−0.033	0.619

PERMANOVA	Sample size	Permutations	Pseudo‐F	*R* ^2^	*p*
Date	18	999	1.505	0.085	0.083
Treatment	18	999	1.518	0.176	0.062

Pairwise	Sample size	Permutations	Pseudo‐F	*R* ^2^	*p*
Control—Humidity	12	999	2.018	0.16	**0.032**
Control—Heat	12	999	1.881	0.158	0.071
Humidity—Heat	12	999	0.088	0.080	0.601

*Note:* Significant results (*p* < 0.05) are in bold.

The clustering in the NMDS plot confirmed the results from the PERMANOVA and ANOSIM analyses, showing that the bacterial community in the Control group is slightly separated from the other groups (Figure 1A). The stress value (0.167) indicates that the NMDS plot is a good representation of the data. Figure 1B shows a high percentage of overlapping regions in the Venn diagram that are common across all treatments (661 ASVs, 31%), suggesting that a substantial proportion of ASVs were shared among treatments. The lower number of shared ASVs corresponded to the overlaps between Control and Humidity (6%) and Humidity and Heat (8%).

#### 3.1.3 Abundance Differences

**FIGURE 2:**
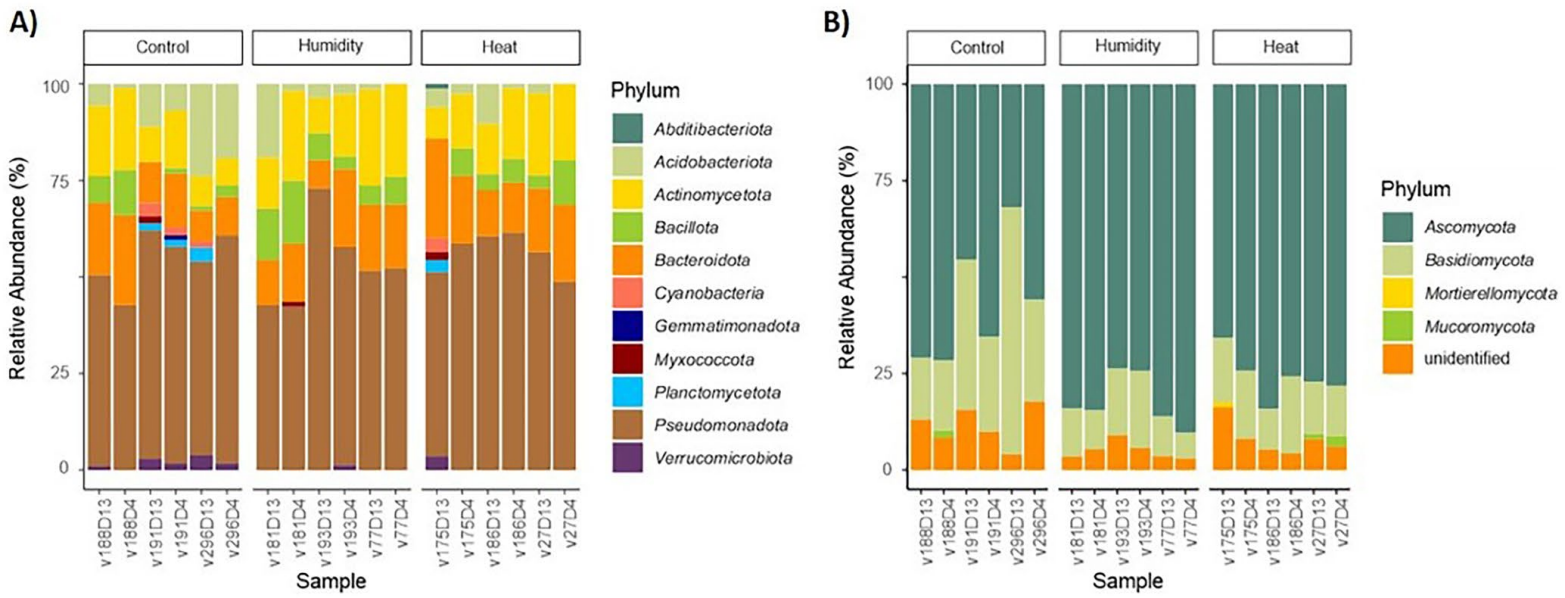
Relative abundances of phylum‐level sequences grouped by treatment (only those with relative abundances > 1% represented in at least one sample). (A) Bacterial sequences; (B) Fungal sequences. The nest identification number and the moment of sampling (at Day 4 or Day 13 of nestling age) corresponding to each sample are shown at the bottom of the figure.

We obtained 1,655,094 high‐quality bacterial sequences, grouped into 12,866 ASVs, which were classified into 2417 species. See Figure S1 and Table S3 for detailed information on the most abundant ASVs classified at the genus level. All samples were dominated by the phylum Pseudomonadota (~Proteobacteria), with percentages ranging from 37% to 70%. Other major bacterial phyla correspond to Bacteroidota (~Bacteroidetes) (7.05%–25.35%), Actinomycetota (~Actinobacteria) (6.94%–24.01%), Bacillota (~Firmicutes) (0.30%–15.78%), Acidobacteriota (0.31%–18.90%) and Verrucomicrobiota (0.16%–3.73%) (Figure 2A). Overall, visual inspection of relative abundance plots did not reveal any major differences between treatments. However, in the control samples, there seems to be a higher percentage of Verrucomicrobiota and Acidobacteriota, while in the samples from heat and humidity treatments, there is a higher percentage of Bacillota (Figure 2A).

**FIGURE 3:**
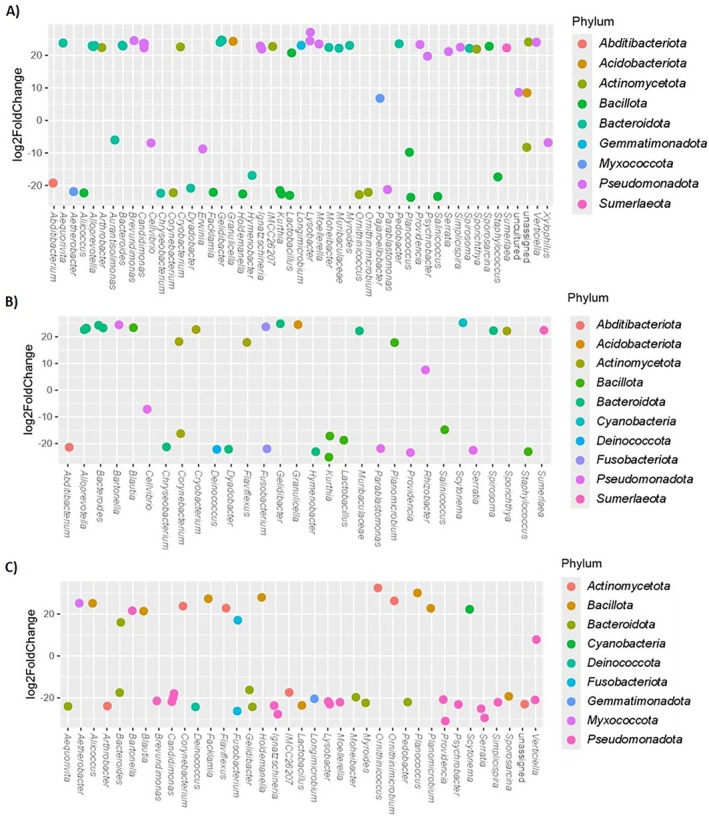
Differential abundance analysis in bacterial sequences, based on *DESeq2* results. The plot shows differences between treatments: (A) Control vs. Humidity, (B) Control vs. Heat and (C) Humidity vs. Heat. In (A, B) a positive log2 fold change value indicates bacteria more abundant in the control condition, while in (C), it indicates bacteria more abundant in the humidity condition. A negative log2 fold change indicates bacteria more abundant in the heat condition in (A), in the heat condition in (B) and in the heat condition in (C). Each point represents ASVs classified at the genus level on the X‐axis. Colours are associated with phyla.

The following differences between bacterial ASVs were found using DESeq2 analysis: 23 ASVs with significantly different abundances between control and heated nests, 52 ASVs with significantly different abundances between control and humidified nests, and 37 ASVs with significantly different abundances between heated and humidified nests (Figure 3; Figure S1; Table S4). Among the most abundant ASVs (those with percentages higher than 1% in some of the samples), differences were observed in the genera *Granulicella*, *Rhizobacter, Lysobacter* and an unidentified member of the family *Caulobacteraceae* which were more abundant in the control nests. On the other hand, *Hymenobacter*, *Chryseobacterium* and *Kurthia* were less abundant in control nests when compared with both humidity and heat treatments. Other abundant genera showed significant relative abundances between treatments. Thus, *Ignatzschineria* was more abundant in the heated nests but was not represented in the samples from the humidified nests. *Bartonella* and *Scytonema* were more abundant in control nests but were not represented in the samples from the heated nests (Figure 3).

#### 3.1.4 Functional Assignment

Functional prediction analysis of bacteria using FAPROTAX software has been able to assign at least one function to 60%–70% of the sequences. Most of them are chemoheterotrophic bacteria (26%–28%), fermenters (0%–5%), capable of degrading urea (2%–9%) and parasites (0.3%–3%) (Figures S2 and S3). The abundance of potentially pathogenic bacteria was not different between treatments (GLMM, *F*_2,14_ = 0.704, *p* = 0.511) and dates (GLMM, *F*_1,14_ = 0.001, *p* = 0.980). See Table S6 for more information on the different percentages of potentially pathogenic bacteria in the different treatments.

### 3.2 Bird Condition and Parasites in Relation to Nest Bacterial Community

The Mantel test showed a significant positive correlation between the bacterial community and the abundance of biting midges and blowflies (Table 3). However, there were no significant correlations between the bacterial community and the rest of the parasites, the nestling body mass, or the body condition of the nestlings (Table 3). The envfit analysis suggests that blowflies have a significant relationship with the ordination of the bacterial community (*p* < 0.05 in the permutation test). In this case, the blowflies vector pointing towards the control group suggests a stronger association between blowfly presence and the bacterial communities in the control group (Figure 1A).

**TABLE 3:** Results of Mantel test relating nest bacterial community and ectoparasite abundance, body mass and body condition of nestlings.

Variable	*r*_s_	*p*
Black flies	0.12	0.093
Biting midges	0.21	**0.022**
Mites	0.04	0.378
Flea larvae	0.07	0.261
Blowflies	0.41	**0.001**
Nestling body mass	−0.13	0.871
Nestling body condition	0.02	0.399

*Note:* Correlation coefficients (*r*_s_) correspond to Spearman correlations. Significant results (*p* < 0.05) are in bold.

Multiple correlation analysis showed a positive and significant association between mites and the bacterial genus *Bartonella* (*r*_s_ = 0.95, *p* < 0.001), between biting midges and the bacterial genus *Ignatzschineria* (*r*_s_ = 0.54, *p* = 0.02), and a negative and significant correlation between nestling body mass and the following bacterial genera: *Pilimelia columellifera* , *Salinimicrobium*, *Acinetobacter*, *Holdemanella*, *Solirubrobacterales* and *Anaerostipes* (*r*_s_ = −0.06, *p* = 0.01, in all cases).

### 3.3 Diversity and Abundance of Fungi

#### 3.3.1 Alpha Diversity

**FIGURE 4:**
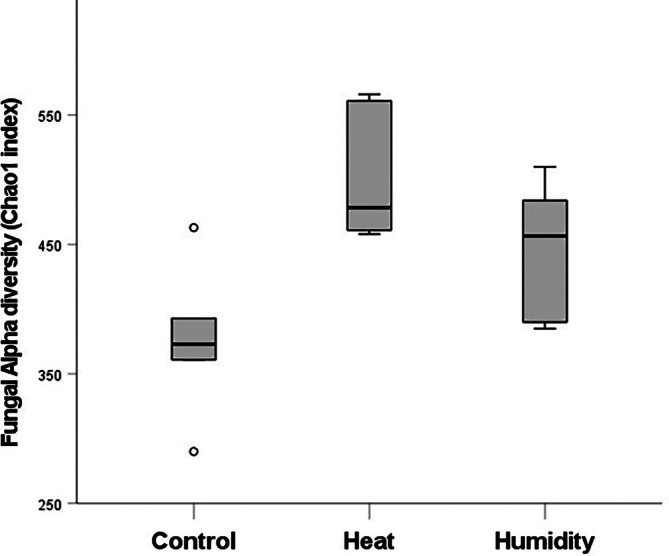
Mean values of fungal alpha diversity according to the Chao1 index depending on the treatment. Median, interquartile range (IQR) and whiskers showing the maximum and minimum values are shown. Individual points outside this range are outliers.

Fungal alpha diversity differs significantly between treatments according to the Chao1 index and is close to significance according to the Shannon index, but not in the case of Simpson's index (Chao1 index: *F* = 8.58, *p* = 0.003; Shannon index: *H* = 5.660, *p* = 0.058; Simpson index: *H* = 3.824, *p* = 0.147). Post hoc analyses showed that diversity is higher in heated than in control nests (Chao1 index: MD = 125.0, *p* = 0.002; Shannon index: MD = −2.37, *p* = 0.026) while there were no significant differences between humidity and control and heated nests (*p* > 0.05 in all cases) (Figure 4). However, there were no significant differences between sampling dates (Shannon index: *W* = 39, *p* = 0.931; Simpson index: *W* = 48, *p* = 0.545; Chao1 index: *t* = −0.24, *p* = 0.81). See Table S5 for more information on the alpha diversity indices in the different treatments.

#### 3.3.2 Beta Diversity

**FIGURE 5:**
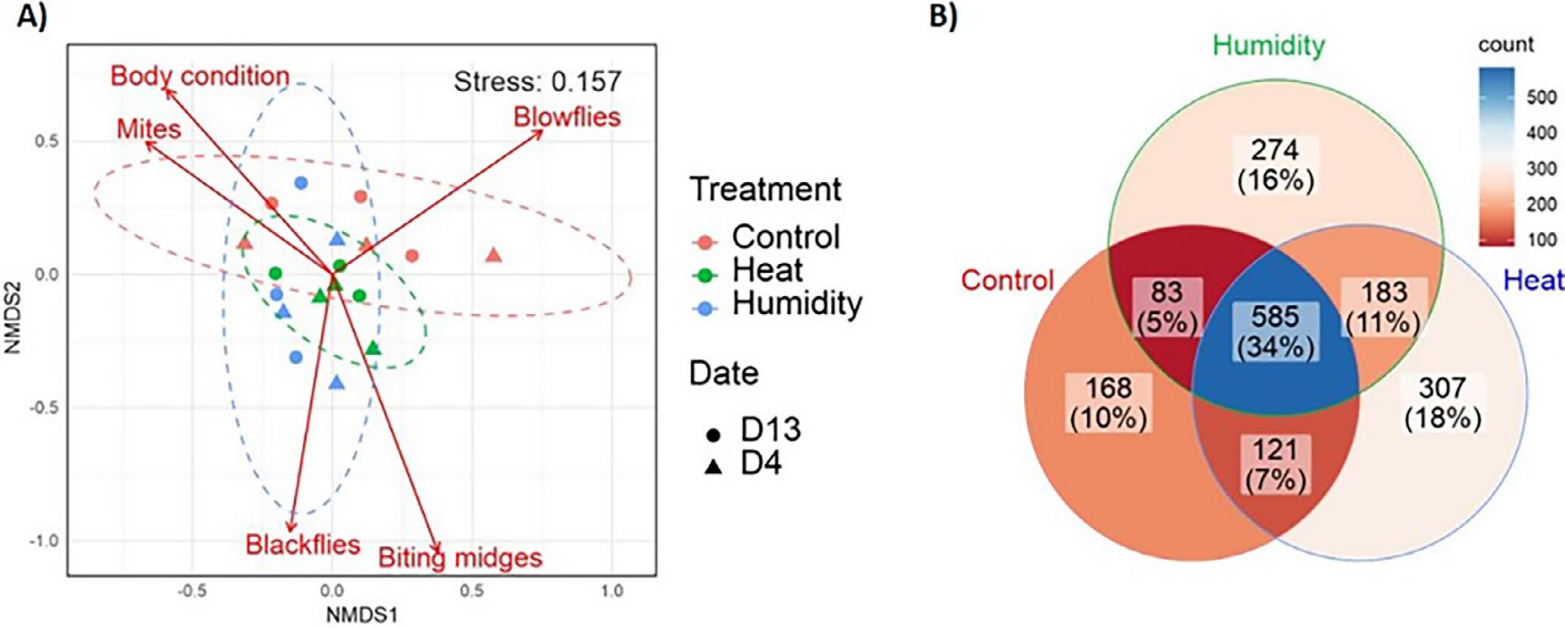
Similarity of the fungal community (Beta diversity). (A) Non‐metric multidimensional scaling (NMDS) ordination plot. The points are individual samples positioned based on their similarity in fungal community composition. The ellipses represent the groupings or clusters based on the variable ‘Treatment’ and show the area where 95% of the points within a group are expected to lie. Red arrows indicate variables fitted to the NMDS ordination using envfit. Each arrow points in the direction of increasing values for that variable. (B) Venn Diagram showing differences or similarities in ASVs at the species level. Each set represents a treatment and the overlap between sets shows which ASVs are shared between them.

Significant differences have been observed in terms of the similarity of the fungal community, specifically between control nests compared to heated and humidified nests (Table 4; Figure 5). However, no differences were found between heated and humidified nests and between sampling dates (Table 4; Figure 5). NMDS shows that the control group is positioned more separately from the heat and humidity groups, while heat and humidity show some overlap, suggesting they are more similar to each other. The stress value (0.157) indicates an acceptable fit for NMDS, meaning the ordination adequately represents the underlying dissimilarity between samples. The Venn diagram shows that the largest overlap (585 ASVs, 34%) is shared among all three treatments, while the smallest overlaps are between Control and Heat (5%) and Control and Humidity (7%) (Figure 5).

**TABLE 4:** Differences in the fungal community (Beta Diversity) between treatments and sampling date according to ANOSIM and PERMANOVA analyses.

ANOSIM	Sample size	Permutations	*R*	*p*
Date	18	999	0.035	0.212
Treatment	18	999	0.137	**0.015**

Pairwise	Sample size	Permutations	*R*	*p*
Control—Humidity	12	999	0.198	**0.016**
Control—Heat	12	999	0.191	**0.020**
Humidity—Heat	12	999	0.041	0.312

PERMANOVA	Sample size	Permutations	Pseudo‐F	*R* ^2^	*p*
Date	18	999	1.088	0.063	0.289
Treatment	18	999	1.534	0.170	**0.007**

Pairwise	Sample size	Permutations	Pseudo‐F	*R* ^2^	*p*
Control—Humidity	12	999	1.692	0.145	**0.008**
Control—Heat	12	999	1.881	0.153	**0.015**
Humidity—Heat	12	999	1.041	0.094	0.389

*Note:* Significant results (*P* < 0.05) are in bold.

#### 3.3.3 Abundance Differences

We obtained 2,092,109 high‐quality fungal sequences, grouped into 8449 ASVs, which were classified into 1942 species. See Figure S4 and Table S7 for detailed information on the most abundant ASVs classified at the genus and species levels. The highest percentage of the ASVs obtained corresponds to the phyla Ascomycota (ranging from 31.76% to 90.11%) and Basidiomycota (ranging from 6.62% to 63.96%) (Figure 2B). The most abundant Ascomycota were associated with the genera *Cladosporium*, *Taeniolella*, *Alternaria*, *Trichocladium*, *Phialemonium*, *Onygena*, *Knufia*, *Staurosphaeria*, *Foliophoma*, *Cladophialophora*, *Penicillium* and *Cryptococcus*. The presence of Basidiomycota genera *Deconica*, *Sistotrema*, *Trechispora*, *Tausonia* and *Cutaneotrichosporon* is also noteworthy. A visual inspection revealed an increase in sequences related to the phylum Ascomycota in samples from heated and humidified nests (Figure 2B).

**FIGURE 6:**
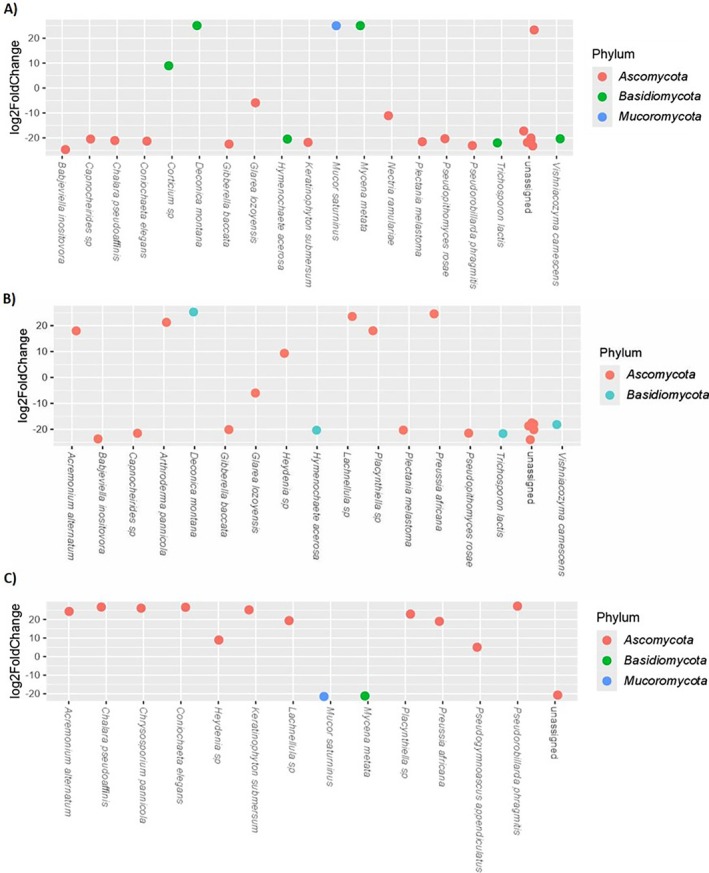
Differential abundance analysis in fungal sequences, based on *DESeq2* results. The plot shows differences between treatments: (A) Control vs. Humidity, (B) Control vs. Heat and (C) Humidity vs. Heat. In (A, B) a positive log2 fold change value indicates fungi more abundant in the control condition, while in (C), it indicates fungi more abundant in the humidity condition. A negative log2 fold change indicates fungi more abundant in the humidity condition in (A), in the heat condition in (B) and in the heat condition in (C). Each point represents ASVs classified at the species level on the X‐axis. Colours are associated with phyla.

The following differences between fungal ASVs were found using DESeq2 analysis: 21 ASVs with significantly different abundances between control and heated nests, 24 ASVs with significantly different abundances between control and humidified nests, and 14 ASVs with significantly different abundances between heated and humidified nests (Figure 6). Among the most abundant ASVs, differences were observed in *Cladosporium, Taeniolella, Trechisporales* and *Trechispora subsphaerospora*, with the genera *Cladosporium* and *Taeniolella* being more abundant in humidity and heated nests, and the order *Trechisporales* and the species *Trechispora subsphaerospora* being more abundant in control nests (Figure S4). In other less abundant ASVs, differences were found between groups, *Arthroderma pannicola* and *Keratinophyton submersum* (~*Chrysosporium submersum*) were more abundant in humidified nests (Figure 6).

#### 3.3.4 Functional Assignment

**FIGURE 7:**
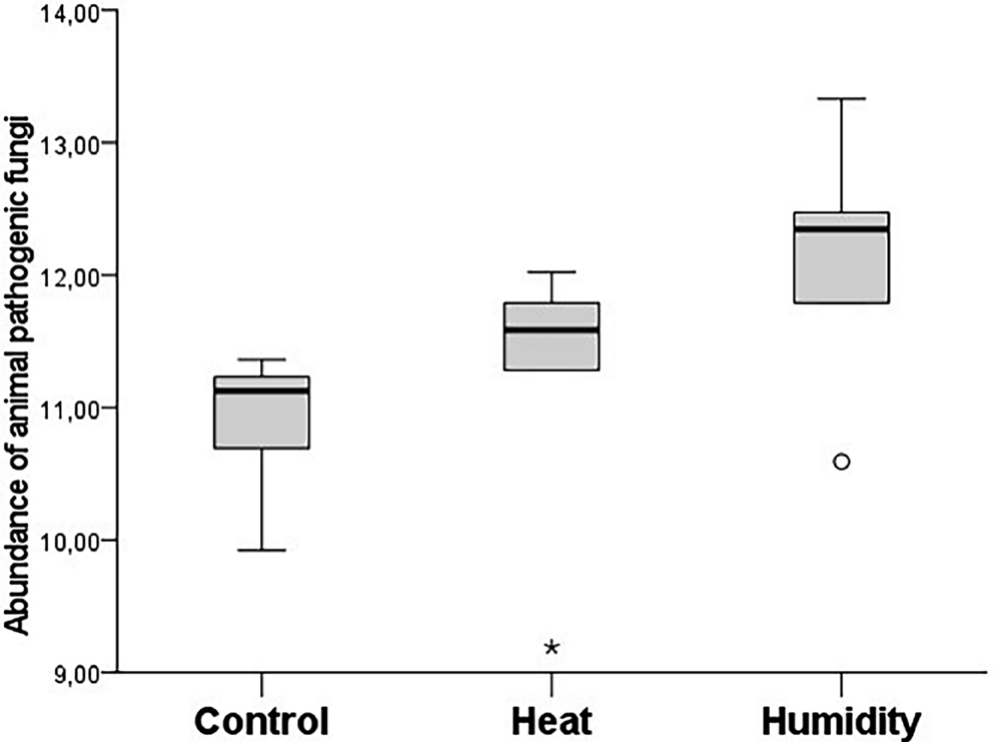
Abundances of potentially pathogenic animal‐associated fungi based on FUNGuild classified by treatment. Median, interquartile range (IQR) and whiskers showing the maximum and minimum values are shown. Individual points outside this range are outliers. The asterisk shows an extreme case.

Functional prediction analysis of fungi using the FUNGuild database classified the majority as saprophytic fungi, with mean abundances ranging from 53.56% to 60.09%, followed by pathotrophic fungi (ranging from 34.56% to 41.96%) and symbiotrophic fungi (ranging from 21.03% to 27.44%) (Figure S5). Among potentially pathogenic fungi, animal pathogenic fungi are of particular interest, with mean abundances ranging from 9.19% to 13.33%. The abundance of animal pathogenic fungi was different between treatments (GLMM, *F*_2,14_ = 12.403, *p <* 0.001), regardless of the date (GLMM, *F*_1,14_ = 0.465, *p* = 0.506). Specifically, they were significantly more abundant in humidified nests as compared to control and heated nests (*t* = 4.930, *p <* 0.001; *t* = 3.076, *p* = 0.008; respectively). However, there were no significant differences between control and heated nests (*t* = −1.855, *p* = 0.085) (Figure 7). See Table S8 for more information on the potentially pathogenic fungi with higher percentages in humidified nests.

### 3.4 Bird Condition and Parasites in Relation to Nest Fungal Community

The Mantel test also showed that there is a significant positive correlation between the fungal community and blowflies, biting midges and blackflies (Table 5). However, there was no significant correlation between the fungal community and the rest of the parasites, the nestling body mass, or the body condition of the nestlings (Table 5). The envfit analysis suggests that biting midges, blackflies, nestling body condition, blowflies and mites have a significant relationship with the ordination of the fungal community (*p* < 0.05 in the permutation test) (Figure 5A). Figure 5A suggests a positive association between the vectors for biting midges and blackflies and the fungal community in the humidified nests, while the vectors for blowflies, mites and nestling body condition point towards control samples, implying a positive association with the fungal community under these conditions.

**TABLE 5:** Mantel test results between nest fungal community and ectoparasite abundance, body mass and body condition of nestlings.

Variable	*r*_s_	*p*
Black flies	0.17	**0.030**
Biting midges	0.25	**0.038**
Mites	0.16	0.177
Flea larvae	−0.02	0.509
Blowflies	0.54	**0.003**
Nestling body mass	−0.07	0.649
Nestling body condition	0.14	0.166

*Note:* Correlation coefficients (*r*_s_) correspond to Spearman correlations. Significant results (*P* < 0.05) are in bold.

Multiple correlation analysis showed a positive and significant correlation between the abundance of the fungal species *Arthroderma melis* and the presence of mites (*r*_s_ = 0.69, *p* < 0.01) and the abundance of *A. pannicola* with the presence of flea larvae (*r*_s_ = 0.71, *p* < 0.01). It also revealed a significant negative correlation between the nestling body condition and *Monographella nivalis* (*R* = −0.61, *p* < 0.01) and the nestling body mass and *Apiotrichum dulcitum* (*R* = −0.66, *p* < 0.01), *Calycina* sp. (*R* = −0.60, *p* = 0.01), *Keratinophyton submersum* (*R* = −0.60, *p* = 0.01), *Tranzsscheliella williamsii* (*R* = −0.60, *p* = 0.01) and *Thyronectria* (*R* = −0.60, *p* = 0.01).

## 4 Discussion

In this study, we conducted a temperature and humidity manipulation experiment inside nest boxes occupied by Eurasian Blue tits. Increases in temperature and humidity negatively affected nestling body condition and the abundance of blowfly larvae. In addition, increased humidity also had a negative effect on mite abundance (see García‐del Río et al. 2025 for further details on these results). We also found that increased temperature and humidity affected the bacterial community, as well as fungal species richness and community composition within the nests. These results provide preliminary evidence that the nest microbiome is influenced by nest microclimate, with differing effects observed between bacteria and fungi. Overall, bacterial communities appeared more resilient to changes in temperature and humidity, as species richness varied little among treatments, despite shifts in community composition under increased humidity. In contrast, fungal communities were more sensitive to the manipulation of both abiotic factors, showing changes in both species' richness and composition.

Furthermore, microclimatic alterations influenced the relationships among microbes, nestlings and ectoparasites within the nests. We identified strong associations between certain bacterial and fungal taxa and ectoparasites, which may facilitate microbial proliferation through the effects of parasitism. Positive relationships between microbial abundance, ectoparasite presence and nestling condition were primarily detected in nests where the nestlings had better body condition and ectoparasites remained abundant or were not negatively affected by microclimatic changes, suggesting that these conditions favoured the development and persistence of coexisting microbial communities. By contrast, such associations were absent in nests where ectoparasite abundance and nestling body condition were reduced.

Another consequence of manipulating the nest microclimate was the proliferation of potentially pathogenic fungi in nests exposed to increased humidity. This may explain why nestlings reared in the more humid nests exhibited poorer body condition, suggesting that elevated humidity may promote microbial growth with potentially detrimental effects on host health and development.

The implications of these findings, together with the remaining results of the study, are discussed in greater detail below.

### 4.1 Diversity and Abundance of Bacteria

The experimental manipulation of nest microclimate and sampling date did not affect bacterial alpha diversity. However, we found that bacterial beta diversity was affected by the experimental manipulation of the nest microclimate. In particular, bacterial beta diversity was significantly different between control and humidified nests. Therefore, the increase in humidity resulted in a change in species composition. This is partly consistent with the results previously obtained in two species of wild passerine birds, the eastern bluebird ( *Sialia sialis* ) and the tree swallow ( *Tachycineta bicolor* ), in which the experimental increase in nest temperature did not influence the alpha diversity of the gut microbiota of nestlings; however, the temperature influenced the beta diversity of eastern bluebird nestling microbiotas (Ingala et al. 2021).

We found that the most abundant bacteria were those corresponding to the phyla Pseudomonadota, Bacteroidota, Actinomycetota, Bacillota, Acidobacteriota and Verrucomicrobiota, in line with the previous studies of nest microbiota (Goodenough and Stallwood 2010; Costanzo et al. 2022; Xin et al. 2023; Yang et al. 2025). Although the differences between treatments were not very evident upon visual inspection, we observed that in heated and humidified nests there was a higher percentage of Bacillota, while in control nests there was a higher percentage of Verrucomicrobiota and Acidobacteriota. Chthoniobacteraceae, a Verrucomicrobiota family identified in various habitats (Granada et al. 2019), primarily soils, showed higher relative abundances in control nests in our study (Figure 5). Similarly, the genus *Granulicella*, one of the most abundant Acidobacteriota in our study with higher relative abundances in control nests, has been isolated from various forest‐related environments (Männistö et al. 2012). Within the phylum Bacillota, the family Planococcaceae, which inhabits a variety of environments and the family Staphylococcaceae, associated with saline environments and skin and mucous membranes (Madigan et al. 2021), were among the most abundant in heated and humidified nests. Most of these bacteria are widely distributed in natural environments, so their presence seems to be associated with the nest material. However, other more abundant bacteria that did not show differences between nest groups, such as Pseudomonadota, include species that can be considered opportunistic pathogens and may have negative effects on the hosts (Shin et al. 2015; Guan et al. 2018). In addition, bacterial composition at the genus level varied between experimental groups. Most were classified as chemoheterotrophic bacteria, which appear to be primarily environmental bacteria with minimal or no evidence of pathogenicity in birds. However, we also identified some ASVs associated with parasitic species. For example, the genus *Facklamia* has been reported to produce septicaemia and meningitis in chickens (Li et al. 2022) and exhibited greater abundance in humidified nests compared to control and heated nests. The genus *Fusobacterium*, known for its zoonotic significance and negative effects on the host (Pandit et al. 2018), was more abundant in heated nests than in control and humidified nests. The genus *Bartonella*, which causes bartonellosis and is transmitted by blood‐feeding arthropods (Mascarelli et al. 2014; Glowska et al. 2020), showed greater abundance in control and humidified nests compared to heated nests. Similarly, the *Serratia* genus, an opportunistic pathogen in passerines (Fudge 2001), was more abundant in heated nests than in control nests. Finally, the genus *Corynebacterium*, occasionally reported as a causative agent of disease in birds (Fiennes 1982), exhibited greater abundance in humidified nests than in control nests. Overall, while some potentially pathogenic bacteria showed greater abundance in specific experimental conditions (e.g., *Facklamia* in humidified nests and *Fusobacterium* in heated nests), functional predictions made using FAPROTAX indicate that this type of bacteria was not influenced by the experiment. Consequently, the poorer condition of the nestlings in these nests does not appear to be associated with an increase in the abundance of potentially pathogenic bacteria.

### 4.2 Diversity and Abundance of Fungi

The experimental manipulation of nest microclimate affected fungal diversity. Alpha diversity was higher in heated nests compared to control nests, regardless of the sampling date. Beta diversity also differed significantly, with distinct species compositions observed between control nests and both humidity and heated nests. Therefore, the temperature rise increased the number of fungal species, and the independent increase in temperature and humidity resulted in a change in species composition. This aligns with the established understanding that fungal growth is promoted by increasing temperatures (Damialis et al. 2015) and by increasing humidity (Bonner and Fergus 1960).

We found that the most abundant fungi were those corresponding to the phyla Ascomycota and Basidiomycota, consistent with previous studies (Xin et al. 2023; Yang et al. 2025). Differences were found between treatments, in particular, increasing humidity and temperature caused an increase in sequences associated with the Ascomycota phylum in these nests. Of particular interest was *Cladosporium*, the most abundant Ascomycota, in which differences were observed. *Cladosporium* is a cosmopolitan genus of fungi found in a wide range of habitats and is commonly present in indoor environments, especially in areas with high humidity and temperature (Katial et al. 1997). In addition, fungal composition at the genus level varied between experimental groups, especially between control nests and heated and humidified nests. Most of the fungi were identified as saprophytes that commonly appear in soils and are associated with the rhizosphere, have specific interactions with plants (phytopathogens or lichens) or insects (entomopathogens), so their impact on birds may be minimal or non‐existent. However, a small proportion of fungi that could be related to bird diseases has been detected (Cannon and Kirk 2007). Particularly, *Candida albicans* , responsible for causing candidiasis in birds, was found in control nests. *Cryptococcus neoformans* , which can infect the central nervous system of wild birds (Bertout et al. 2022), the genus *Rhodotorula*, responsible for causing rhodotoruliasis in birds and the species *Mucor racemosus* and *Mucor circinelloides*, which are opportunistic fungi and can cause serious infections in birds with compromised immune systems, were found predominantly in heated and humidified nests (Malik et al. 2021; Bertout et al. 2022). In addition, sequences related to dermatophyte fungi have been found: *Arthroderma pannicola* and *Keratinophyton submersum* (or *Chrysosporium submersum*), which were more abundant in humidified nests. These fungi are found in soils and organic waste, decomposing keratin and playing an ecological role in nutrient recycling, as well as being an opportunistic pathogen in dermatophytosis in humans and animals (Ulfig 1994; Labuda et al. 2021). Keratinolytic fungi could have an important impact on young birds during feather production (i.e., the period when altricial birds are in the nest), as feather‐degrading microbes can potentially reduce bird fitness (Goodenough and Stallwood 2010). Notably, fungi classified as animal pathogens were more abundant in humidified nests, potentially accounting for the poorer body condition of the nestlings observed in these nests (García‐del Río et al. 2025). Therefore, these effects may be interrelated, as elevated humidity promotes the proliferation of potentially pathogenic dermatophytic fungi, which could subsequently have a detrimental impact on nestlings. This is particularly significant given the nest's central role in the assembly of the nestling microbiome (Campos‐Cerda and Bohannan 2020).

### 4.3 Bird Condition and Parasites in Relation to Nest Bacterial Community

We found a significant positive correlation between the composition of the bacterial community and the abundance of biting midges and blowflies. Other studies have found associations between these ectoparasites and bacterial communities and shown that these communities can be affected by factors such as temperature and humidity (Bennett et al. 2019; Möhlmann et al. 2022; Neupane et al. 2023). Furthermore, the abundance of blowflies exhibited a significant relationship with the ordination of the bacterial community, with a notably stronger association between blowfly abundance and bacterial communities in the control group. This aligns with the finding that these nests exhibited the highest abundance of these ectoparasites (García‐del Río et al. 2025). However, we did not find a significant correlation between the body condition of the nestlings and the bacterial community.

On the other hand, we found a positive correlation between mites and the bacterial genus *Bartonella*, consistent with the fact that these avian‐associated arthropods are known to carry *Bartonella* spp. (Hubert et al. 2017). We also found a positive correlation between biting midges and the bacterial genus *Ignatzschineria*, which has species associated with the presence of insects and their larvae (Iancu et al. 2020), and a negative correlation between nestling body mass and several bacterial genera, including *Acinetobacter*, which has been associated with skin and mucous diseases in birds (Liu et al. 2016; Lupo et al. 2018).

### 4.4 Bird Condition and Parasites in Relation to Nest Fungal Community

We found a positive correlation between the fungal community and the abundance of blowflies, biting midges and blackflies. Furthermore, the abundance of biting midges, blackflies, blowflies, mites and the nestling body condition exhibited a significant relationship with the ordination of the fungal community. In particular, we found a positive association between the abundance of biting midges and blackflies and the fungal community in humidified nests. The direction and length of the biting midges and blackflies vectors in Figure 3A indicate that higher humidity levels could be promoting specific fungal taxa that coexist with these ectoparasites. Also, we found a positive association between the abundance of blowflies, mites, nestling body condition and the fungal community in control nests. This suggests that control nests, characterised by a higher ectoparasite abundance and improved nestling body condition, provided favourable conditions for specific fungal taxa that coexist with these ectoparasites and nestlings.

On the other hand, we found a positive correlation between the abundance of the fungus species *Arthroderma melis* and the presence of mites, and between the abundance of *Arthroderma pannicola* and the presence of flea larvae. Since both species are keratinolytic fungi, the presence of these ectoparasites infesting the nestlings could be beneficial for them, as the remnants of skin produced by injuries might promote the colonisation and growth of bacteria (Ulfig 1994; Weiss 2006; Labuda et al. 2021). Also, we found a negative correlation between nestling body condition and *Monographella nivalis*, as well as a negative correlation between nestling body mass and *Apiotrichum dulcitum*, *Calycina* sp., *Keratinophyton submersum*, *Tranzsscheliella williamsii* and *Thyronectria* sp. These fungi generally have no functions associated with birds, except for *K. submersum*, which is a ubiquitous and keratinolytic species. This genus is particularly common, especially in areas with high animal activity, where abundant keratinous material is available (Papini et al. 1998; Labuda et al. 2021).

This study demonstrated that nest environmental conditions significantly shape its microbiome and revealed its relationship with the abundance of ectoparasitic arthropods and nestling development. Additionally, our findings suggest a complex interplay among these factors, as evidenced by the proliferation of potentially pathogenic dermatophytic fungi in nests where the microclimate was experimentally manipulated, with relative humidity emerging as the most influential parameter. However, the use of the gel in the humidity treatment could represent a limitation, as it introduces nutrients that could influence microbial communities. Notably, this study provides the first experimental analysis of microclimate effects on the nest microbiome. These insights enhance our understanding of how environmental shifts within nests impact microbial communities and their interactions with nestling growth and associated parasites. However, the results should be interpreted with caution due to the low sample size and repeated sampling of the same nests, which may limit the statistical power and the generalisation of the findings. Although nest identity was accounted for in mixed models, ordination analyses and Mantel tests should be interpreted with caution, as they are used as exploratory and complementary tools to describe potential associations. In addition, the causal direction of the relationships among microclimate, microbiome, parasites and nestling condition remains unclear, as it is not possible to determine whether it is a direct or indirect effect of the microclimate on all or some of the variables under study. Experimental manipulation of bacterial and fungal communities or parasite loads would be necessary to disentangle the underlying mechanisms behind these relationships. These findings underscore the need to further investigate the relationship between broader forest climatic conditions and nest microenvironments, as these factors may be critical in shaping brood development.

## Author Contributions

**Marina García‐del Río:** formal analysis (equal), investigation (equal), methodology (equal), writing – original draft (equal). **Tamara Martin‐Pozas:** data curation (equal), methodology (equal) formal analysis (equal), writing – original draft (equal). **Sergio Sanchez‐Moral:** conceptualization (equal), writing – review and editing (equal). **Alejandro Cantarero:** methodology (equal), writing – review and editing (equal). **Francisco Castaño‐Vázquez:** methodology (equal). **Yago Merino:** methodology (equal). **Javier García‐Velasco:** methodology (equal). **Santiago Merino:** conceptualization (equal), funding acquisition (equal), methodology (equal), project administration (equal), writing – review and editing (equal).

## Funding

This work was supported by the Ministerio de Ciencia, Innovación y Universidades (MICIU/AEI/10.13039/501100011033) [Grant numbers PID2023‐149436NB‐I00, RYC2022‐035559‐I and JDC2023‐051909‐1]; the European Regional Development Fund (ERDF) ‘A way of making Europe’; and the European Social Fund Plus (ESF+).

## Ethics Statement

This study complies with current European legislation on experimental procedures with animals (2010/63/UE) and was reviewed and approved by the Dirección General de Agricultura, Ganaderia y Alimentación, Comunidad de Madrid (Spain), Permission PROEX 128/19. Annual ringing permissions were provided by Junta de Castilla y León.

## Conflicts of Interest

We disclose any actual or potential conflicts of interest including any financial, personal, or other relationships with other people or organisations that could inappropriately influence, or be perceived to influence, our work.

## Supporting information


**Table S1:** Results of extraction (Bex) and PCR blanks (Bpcr).
**Table S2:** Alpha diversity indexes of bacterial communities.
**Table S4:** Results of the DESeq analysis for the bacterial communities, grouped by treatment.
**Table S5:** Alpha diversity indexes of fungal communities.
**Table S6:** Classification of potentially pathogenic bacteria, parasites and symbionts based on FAPROTAX.
**Table S8:** Classification of potentially pathogenic animal‐associated fungi with higher relative abundance in humidified nests based on FUNGuild analysis.
**Figure S1:** Relative abundances of bacterial sequences at genus level (only those with relative abundances > 1% are represented).
**Figure S2:** Relative abundance of bacteria with relative abundances > 15% grouped by their main ecological functions and by treatment based on FAPROTAX.
**Figure S3:** Relative abundance of bacteria with relative abundances between 1% and 15% grouped by their main ecological functions and by treatment based on FAPROTAX.
**Figure S4:** Relative abundances of fungal sequences at species level (only those with relative abundances > 2% are represented).
**Figure S5:** Relative abundance of fungi grouped by their main ecological functions and by treatment based on FUNGuild.

**Table S3:** Relative abundance of bacteria at the genus level according to treatment.

**Table S7:** Relative abundance of fungi at the genus level according to treatment.

## Data Availability

The gene sequences and metadata from this study were deposited in the Sequence Read Archive (SRA) of NCBI under the BioProject ID PRJNA1227866.
