# Supplementary material for: Environmental Drivers on Blue Tit Nest Microbiome: An Experimental Study
Source: Ecol Evol. 2026 Jul 14;16(7):e74007. doi: 10.1002/ece3.74007 (PMC13368405; doi:10.1002/ece3.74007)

**Table S1.** Results of extraction and PCR blanks (Extraction blanks: Bex; PCR blanks: Bpcr).

| **OTU ID** | **Taxonomy** | **Bex** | **Bpcr** |
| --- | --- | --- | --- |
| a00842cfc255cd2cc223a7a551ed5662 | k__Fungi; p__Basidiomycota; c__Tremellomycetes; o__Trichosporonales; f__Trichosporonaceae; g__Cutaneotrichosporon; s__Cutaneotrichosporon_debeurmannianum | 380 | 700 |
| 3138b36d9e7461af13845ec8c092452e | k__Fungi; p__Basidiomycota; c__Malasseziomycetes; o__Malasseziales; f__Malasseziaceae; g__Malassezia; s__Malassezia_restricta | 40 | 0 |
| 13d36234f283af901caef9efdaeaac11 | k__Fungi; p__Basidiomycota; c__Microbotryomycetes; o__Sporidiobolales; f__Sporidiobolaceae; g__Rhodotorula; s__Rhodotorula_mucilaginosa | 50 | 0 |
| c77652dceea0e2d31ed0cdefd5331546 | k__Fungi; p__Ascomycota; c__Saccharomycetes; o__Saccharomycetales; f__Saccharomycetales_fam_Incertae_sedis; g__Candida; s__Candida_railenensis | 40 | 0 |
| 813647fe0078fb7d492bec8288c531f6 | k__Fungi; p__Basidiomycota; c__Agaricomycetes; o__Agaricales; f__Omphalotaceae; g__Lentinula; s__Lentinula_edodes | 20 | 0 |
| 5df56b374f11ea94cdf0502f568e90aa | k__Fungi; p__Ascomycota; c__Dothideomycetes; o__Dothideales; f__Saccotheciaceae; g__Aureobasidium; s__Aureobasidium_pullulans | 0 | 130 |
| ff75a588c4e4c84a13302714c9c099ef | k__Fungi; p__Ascomycota; c__Dothideomycetes | 0 | 40 |
| 8d2bbd92081d2992f918c337d7529633 | k__Fungi; p__Ascomycota; c__Leotiomycetes; o__Thelebolales; f__Thelebolaceae; g__Thelebolus; s__Thelebolus_globosus | 0 | 70 |
| 92cd5bcf8f7caa2304a8ae1d0ec61ab5 | k__Fungi; p__Ascomycota; c__Dothideomycetes; o__Myriangiales; f__Endosporiaceae; g__Endosporium; s__Endosporium_sp | 0 | 20 |
| a72b9cb91c97847258fcefc70e88ab8e | k__Fungi; p__Ascomycota; c__Leotiomycetes; o__Helotiales; f__Helotiaceae; g__Articulospora | 0 | 40 |
| f52e759cf3521ac900d58666d5f0f54a | d__Bacteria; p__Proteobacteria; c__Gammaproteobacteria; o__Pseudomonadales; f__Moraxellaceae; g__Acinetobacter; s__Acinetobacter_bereziniae | 442 | 0 |
| 3638f2062c305c79df283ef573a10b97 | d__Bacteria; p__Proteobacteria; c__Gammaproteobacteria; o__Burkholderiales; f__Burkholderiaceae; g__Ralstonia | 32 | 0 |
| cc3972883fd7f6156ba7a61c2172183b | d__Bacteria; p__Proteobacteria; c__Alphaproteobacteria; o__Sphingomonadales; f__Sphingomonadaceae; g__Sphingomonas | 11 | 0 |
| e01ccd749960b04699eef783119e25ae | d__Bacteria; p__Proteobacteria; c__Gammaproteobacteria; o__Pseudomonadales; f__Pseudomonadaceae; g__Pseudomonas | 4 | 0 |
| 387349bbf301de818017a263010277fa | d__Bacteria; p__Proteobacteria; c__Gammaproteobacteria; o__Xanthomonadales; f__Xanthomonadaceae; g__Stenotrophomonas | 8 | 0 |
| 0a1f5ed70dcb37a86924dbd5fde981af | d__Bacteria; p__Proteobacteria; c__Gammaproteobacteria; o__Pseudomonadales; f__Moraxellaceae; g__Enhydrobacter | 6 | 0 |
| 75caefa0df8e0699a60f04fa154f0bba | d__Bacteria; p__Proteobacteria; c__Gammaproteobacteria; o__Pseudomonadales; f__Moraxellaceae; g__Acinetobacter | 7 | 0 |
| 4c7a497eca1fa3081f152adbbce602fb | d__Bacteria; p__Proteobacteria; c__Gammaproteobacteria; o__Burkholderiales; f__Comamonadaceae; g__Delftia | 15 | 0 |
| 2a29610f7278db2fa05261fb21fdefca | d__Bacteria; p__Proteobacteria; c__Gammaproteobacteria; o__Burkholderiales; f__Burkholderiaceae; g__Ralstonia | 9 | 0 |
| babc8d2a94de6a53eca8f66aaf4427d9 | d__Bacteria; p__Firmicutes; c__Bacilli; o__Staphylococcales; f__Staphylococcaceae; g__Staphylococcus | 7 | 0 |
| 3bf37d2db9ed7cedef08f606ca3722f8 | d__Bacteria; p__Proteobacteria; c__Alphaproteobacteria; o__Caulobacterales; f__Caulobacteraceae; g__Brevundimonas; s__Brevundimonas_terrae | 4 | 0 |
| 07c3160551922faa7be5740ea3adc189 | d__Bacteria; p__Proteobacteria; c__Gammaproteobacteria; o__Burkholderiales; f__Burkholderiaceae; g__Ralstonia; s__Ralstonia_insidiosa | 0 | 20 |
| be5f7561425c2fdf15e1347047e3a7ba | d__Bacteria; p__Proteobacteria; c__Gammaproteobacteria; o__Pseudomonadales; f__Pseudomonadaceae; g__Pseudomonas | 0 | 5 |

**Table S2.** Alpha diversity indexes of bacterial communities.

| **Nest** | **Treatment** | **Date** | **Observed** | **Chao1** | **Shannon** | **Simpson** |
| --- | --- | --- | --- | --- | --- | --- |
| 175 | Heat | 13 | 415 | 415.0294 | 4.615332 | 0.9797963 |
| 175 | Heat | 4 | 628 | 629.1111 | 5.098643 | 0.9859378 |
| 181 | Humidity | 13 | 558 | 558 | 4.721702 | 0.9729617 |
| 181 | Humidity | 4 | 565 | 565 | 5.105382 | 0.9864968 |
| 186 | Heat | 13 | 595 | 596.4737 | 4.757838 | 0.9807063 |
| 186 | Heat | 4 | 441 | 441.1304 | 4.230726 | 0.9512086 |
| 188 | Control | 13 | 686 | 687 | 5.254447 | 0.988974 |
| 188 | Control | 4 | 570 | 570.5455 | 5.061319 | 0.9863876 |
| 191 | Control | 13 | 498 | 498.1765 | 4.635542 | 0.9755287 |
| 191 | Control | 4 | 587 | 588.5556 | 5.099966 | 0.9878209 |
| 193 | Humidity | 13 | 435 | 435.2308 | 3.762506 | 0.8793715 |
| 193 | Humidity | 4 | 481 | 481 | 4.639376 | 0.9755841 |
| 27 | Heat | 13 | 598 | 598.1429 | 4.962649 | 0.9824571 |
| 27 | Heat | 4 | 472 | 472.4688 | 4.601178 | 0.9760138 |
| 296 | Control | 13 | 418 | 418 | 4.488575 | 0.973236 |
| 296 | Control | 4 | 302 | 302 | 4.018418 | 0.9612223 |
| 77 | Humidity | 13 | 574 | 574.0455 | 4.760752 | 0.9712052 |
| 77 | Humidity | 4 | 453 | 453.04 | 4.206729 | 0.9554261 |

**Table S4.** Results of DESeq analysis for the bacterial communities, grouped by treatment.

| **Control treatment (C) Vs. Heat treatment (H)** | | | | | | | |  |  |  |
| --- | --- | --- | --- | --- | --- | --- | --- | --- | --- | --- |
| **Phylum** | **Class** | **Order** | **Family** | **Genus** | **Species** | **log2FoldChange** | **pvalue** | **C(%)** | **H(%)** | **Source** |
| Abditibacteriota | Abditibacteria | Abditibacteriales | Abditibacteriaceae | Abditibacterium | uncultured | -21.57 | 0.000 | 0.00 | 0.08 | Environment |
| Actinomycetota | Actinobacteria | Actinomycetales | Actinomycetaceae | Flaviflexus | uncultured | 17.23 | 0.000 | 0.00 | 0.00 | Animal |
| Actinomycetota | Actinobacteria | Corynebacteriales | Corynebacteriaceae | Corynebacterium | C. crudilactis | -15.86 | 0.000 | 0.00 | 0.00 | Animal, Env.,Pathogen |
| Actinomycetota | Actinobacteria | Corynebacteriales | Corynebacteriaceae | Corynebacterium | C. marinum | 18.14 | 0.000 | 0.00 | 0.00 | Environment |
| Actinomycetota | Actinobacteria | Micrococcales | Bogoriellaceae | Georgenia | unidentified | 20.22 | 0.000 | 0.01 | 0.00 | Environment |
| Bacillota | Bacilli | Bacillales | Planococcaceae | Kurthia | Kurthia_sp. | -16.75 | 0.000 | 0.00 | 0.01 | Animal |
| Bacillota | Bacilli | Bacillales | Planococcaceae | Lysinibacillus | unidentified | -24.22 | 0.000 | 0.01 | 0.33 | Environment |
| Bacillota | Bacilli | Lactobacillales | Lactobacillaceae | Lactobacillus | L. murinus | -18.44 | 0.000 | 0.00 | 0.02 | Animal gut |
| Bacillota | Bacilli | Staphylococcales | Staphylococcaceae | Salinicoccus | uncultured | -14.55 | 0.000 | 0.00 | 0.00 | Animal, Env. |
| Bacillota | Bacilli | Staphylococcales | Staphylococcaceae | Staphylococcus | S. succinus | -22.88 | 0.000 | 0.00 | 0.17 | Environment |
| Bacillota | Clostridia | Lachnospirales | Lachnospiraceae | Blautia | B. glucerasea | 23.27 | 0.000 | 0.08 | 0.00 | Animal gut |
| Bacteroidota | Bacteroidia | Cytophagales | Hymenobacteraceae | Hymenobacter | H. elongatus | -23.46 | 0.000 | 0.00 | 0.18 | Animal, Env |
| Bacteroidota | Bacteroidia | Flavobacteriales | Flavobacteriaceae | Gelidibacter | unidentified | 23.30 | 0.000 | 0.24 | 0.00 | Environment |
| Bacteroidota | Bacteroidia | Flavobacteriales | Weeksellaceae | Chryseobacterium | C. yonginense | -21.76 | 0.000 | 0.00 | 0.09 | Environment |
| Bacteroidota | Bacteroidia | Flavobacteriales | Weeksellaceae | Empedobacter | E. brevis | 9.02 | 0.001 | 0.27 | 0.01 | Environment |
| Cyanobacteria | Cyanobacteriia | Cyanobacteriales | Nostocaceae | Scytonema_UTEX_2349 | unidentified | 23.71 | 0.000 | 0.71 | 0.00 | Environment |
| Fusobacteriota | Fusobacteriia | Fusobacteriales | Fusobacteriaceae | Fusobacterium | Fusobacterium_sp. | -21.32 | 0.000 | 0.00 | 0.05 | Animal flora, pathogen |
| Pseudomonadota | Alphaproteobacteria | Rhizobiales | Rhizobiaceae | Bartonella | unidentified | 25.47 | 0.000 | 0.39 | 0.00 | Parasite, mite |
| Pseudomonadota | Alphaproteobacteria | Sphingomonadales | Sphingomonadaceae | Parablastomonas | uncultured | -21.99 | 0.000 | 0.00 | 0.11 | Environment |
| Pseudomonadota | Gammaproteobacteria | Cellvibrionales | Cellvibrionaceae | Cellvibrio | unidentified | -7.68 | 0.000 | 0.00 | 0.05 | Environment |
| Pseudomonadota | Gammaproteobacteria | Enterobacterales | Morganellaceae | Providencia | P. alcalifaciens | -22.58 | 0.000 | 0.00 | 0.11 | Animal flora |
| Pseudomonadota | Gammaproteobacteria | Enterobacterales | Yersiniaceae | Serratia | S. plymuthica | -22.76 | 0.000 | 0.00 | 0.09 | Env.,Pathogen |
| Pseudomonadota | Gammaproteobacteria | Pseudomonadales | Pseudomonadaceae | Pseudomonas | P. brenneri | 7.84 | 0.001 | 0.05 | 0.00 | Environment |
| **Control treatment (C) Vs. Humidity treatment (Hum)** | | | | | | | |  |  |  |
| **Phylum** | **Class** | **Order** | **Family** | **Genus** | **Species** | **log2FoldChange** | **pvalue** | **C(%)** | **Hum(%)** | **Source** |
| Abditibacteriota | Abditibacteria | Abditibacteriales | Abditibacteriaceae | Abditibacterium | uncultured | -18.79 | 0.000 | 0.00 | 0.01 | Environment |
| Acidobacteriota | Acidobacteriae | Acidobacteriales | Acidobacteriaceae | Granulicella | uncultured | 25.10 | 0.000 | 0.18 | 0.00 | Environment |
| Acidobacteriota | Acidobacteriae | Acidobacteriales | Acidobacteriaceae | Granulicella | unidentified | 3.56 | 0.003 | 5.34 | 0.98 | Environment |
| Actinomycetota | Acidimicrobiia | Microtrichales | Microtrichaceae | IMCC26207 | unidentified | 21.94 | 0.000 | 0.06 | 0.00 | Environment |
| Actinomycetota | Actinobacteria | Corynebacteriales | Corynebacteriaceae | Corynebacterium | C. crudilactis | -22.99 | 0.000 | 0.00 | 0.15 | Animal, Env.,Pathogen |
| Actinomycetota | Actinobacteria | Micrococcales | Intrasporangiaceae | Ornithinicoccus | uncultured | -22.86 | 0.000 | 0.00 | 0.18 | Environment |
| Actinomycetota | Actinobacteria | Micrococcales | Intrasporangiaceae | Ornithinimicrobium | unidentified | -23.65 | 0.000 | 0.00 | 0.09 | Environment |
| Actinomycetota | Actinobacteria | Micrococcales | Intrasporangiaceae | unidentified | unidentified | -8.42 | 0.000 | 0.00 | 0.14 | Environment |
| Actinomycetota | Actinobacteria | Micrococcales | Micrococcaceae | Yaniella | uncultured | -21.44 | 0.000 | 0.00 | 0.04 | Environment |
| Actinomycetota | Actinobacteria | Micrococcales | unidentified | unidentified | unidentified | 24.36 | 0.000 | 0.15 | 0.00 | Environment |
| Actinomycetota | Actinobacteria | Propionibacteriales | Nocardioidaceae | Nocardioides | N. gilvus | -22.15 | 0.000 | 0.00 | 0.05 | Environment |
| Bacillota | Bacilli | Bacillales | Planococcaceae | Kurthia | Kurthia_sp. | -21.32 | 0.000 | 0.00 | 0.10 | Environment |
| Bacillota | Bacilli | Bacillales | Planococcaceae | Lysinibacillus | unidentified | -17.22 | 0.000 | 0.01 | 0.01 | Environment |
| Bacillota | Bacilli | Bacillales | Planococcaceae | Planococcus | uncultured | -22.77 | 0.000 | 0.00 | 0.25 | Environment |
| Bacillota | Bacilli | Bacillales | Planococcaceae | Planococcus | unidentified | -9.01 | 0.000 | 0.00 | 0.27 | Environment |
| Bacillota | Bacilli | Lactobacillales | Aerococcaceae | Facklamia | unidentified | -21.25 | 0.000 | 0.00 | 0.09 | Animal,pathogen |
| Bacillota | Bacilli | Lactobacillales | Lactobacillaceae | Lactobacillus | L. murinus | -22.98 | 0.000 | 0.00 | 0.28 | Animal gut |
| Bacillota | Bacilli | Staphylococcales | Staphylococcaceae | Aliicoccus | uncultured | -22.96 | 0.000 | 0.00 | 0.12 | Environment |
| Bacillota | Bacilli | Staphylococcales | Staphylococcaceae | Salinicoccus | uncultured | -22.49 | 0.000 | 0.00 | 0.34 | Animal, Env. |
| Bacillota | Bacilli | Staphylococcales | Staphylococcaceae | Staphylococcus | S. succinus | -16.53 | 0.000 | 0.00 | 0.01 | Environment |
| Bacteroidota | Bacteroidia | Chitinophagales | Chitinophagaceae | Aurantisolimonas | unidentified | -5.85 | 0.001 | 0.00 | 0.05 | Environment |
| Bacteroidota | Bacteroidia | Chitinophagales | Chitinophagaceae | uncultured | unidentified | 3.63 | 0.002 | 1.32 | 0.17 | Environment |
| Bacteroidota | Bacteroidia | Cytophagales | Hymenobacteraceae | Hymenobacter | H. elongatus | -17.71 | 0.000 | 0.00 | 0.00 | Environment |
| Bacteroidota | Bacteroidia | Flavobacteriales | Flavobacteriaceae | Aequorivita | A. capsosiphonis | 24.02 | 0.000 | 0.12 | 0.00 | Environment |
| Bacteroidota | Bacteroidia | Flavobacteriales | Flavobacteriaceae | Flavobacterium | uncultured | -7.39 | 0.001 | 0.00 | 0.06 | Environment |
| Bacteroidota | Bacteroidia | Flavobacteriales | Flavobacteriaceae | Gelidibacter | unidentified | 24.81 | 0.000 | 0.21 | 0.00 | Environment |
| Bacteroidota | Bacteroidia | Flavobacteriales | Flavobacteriaceae | Gelidibacter | uncultured | 22.83 | 0.000 | 0.14 | 0.00 | Environment |
| Bacteroidota | Bacteroidia | Flavobacteriales | Flavobacteriaceae | Gelidibacter | unidentified | 23.43 | 0.000 | 0.24 | 0.00 | Environment |
| Bacteroidota | Bacteroidia | Flavobacteriales | Weeksellaceae | Chryseobacterium | C. yonginense | -21.80 | 0.000 | 0.00 | 0.12 | Environment |
| Chloroflexota | Chloroflexotaa | Thermomicrobiales | JG30-KF-CM45 | JG30-KF-CM45 | bacterium_QTYC46b | -21.50 | 0.000 | 0.00 | 0.03 | Environment |
| Gemmatimonadota | Longimicrobia | Longimicrobiales | Longimicrobiaceae | Longimicrobium | unidentified | 23.67 | 0.000 | 0.17 | 0.00 | Environment |
| Planctomycetota | Phycisphaerae | Tepidisphaerales | WD2101_soil_group | WD2101_soil_group | uncultured | 3.76 | 0.001 | 0.42 | 0.05 | Environment |
| Pseudomonadota | Alphaproteobacteria | Acetobacterales | Acetobacteraceae | Acidiphilium | uncultured | 3.31 | 0.002 | 0.87 | 0.20 | Environment |
| Pseudomonadota | Alphaproteobacteria | Acetobacterales | Acetobacteraceae | Acidiphilium | unidentified | 3.19 | 0.001 | 4.82 | 1.02 | Environment |
| Pseudomonadota | Alphaproteobacteria | Caulobacterales | Caulobacteraceae | Brevundimonas | B. diminuta | 24.96 | 0.000 | 0.25 | 0.00 | Plant pathogen |
| Pseudomonadota | Alphaproteobacteria | Caulobacterales | Caulobacteraceae | uncultured | unidentified | 3.76 | 0.000 | 1.48 | 0.19 | Environment |
| Pseudomonadota | Alphaproteobacteria | Rhizobiales | Beijerinckiaceae | 1174-901-12 | uncultured | 2.78 | 0.002 | 2.84 | 0.66 | Environment |
| Pseudomonadota | Alphaproteobacteria | Rhizobiales | Rhizobiaceae | Pararhizobium | P. herbae | -3.75 | 0.001 | 0.01 | 0.20 | Soil |
| Pseudomonadota | Alphaproteobacteria | Sphingomonadales | Sphingomonadaceae | Parablastomonas | uncultured | -20.86 | 0.000 | 0.00 | 0.07 | Environment |
| Pseudomonadota | Gammaproteobacteria | Burkholderiales | Alcaligenaceae | Candidimonas | unidentified | 22.40 | 0.000 | 0.11 | 0.00 | Animal,Env. |
| Pseudomonadota | Gammaproteobacteria | Burkholderiales | Alcaligenaceae | Candidimonas | uncultured | 22.53 | 0.000 | 0.04 | 0.00 | Animal,Env. |
| Pseudomonadota | Gammaproteobacteria | Burkholderiales | Comamonadaceae | Simplicispira | unidentified | 23.04 | 0.000 | 0.06 | 0.00 | Environment |
| Pseudomonadota | Gammaproteobacteria | Burkholderiales | Oxalobacteraceae | Duganella | Duganella_sp. | -7.53 | 0.001 | 0.00 | 0.02 | Environment |
| Pseudomonadota | Gammaproteobacteria | Cardiobacteriales | Wohlfahrtiimonadaceae | Ignatzschineria | Ignatzschineria_sp. | 24.53 | 0.000 | 0.03 | 0.00 | Insects |
| Pseudomonadota | Gammaproteobacteria | Cardiobacteriales | Wohlfahrtiimonadaceae | Ignatzschineria | uncultured | 25.55 | 0.000 | 0.06 | 0.00 | Insects |
| Pseudomonadota | Gammaproteobacteria | Cellvibrionales | Cellvibrionaceae | Cellvibrio | unidentified | -6.60 | 0.000 | 0.00 | 0.05 | Environment |
| Pseudomonadota | Gammaproteobacteria | Enterobacterales | Erwiniaceae | Erwinia | E. billingiae | -8.33 | 0.000 | 0.00 | 0.18 | Plant pathogen |
| Pseudomonadota | Gammaproteobacteria | Enterobacterales | Morganellaceae | Providencia | P. heimbachae | 23.69 | 0.000 | 0.10 | 0.00 | Animal gut |
| Pseudomonadota | Gammaproteobacteria | Xanthomonadales | Xanthomonadaceae | Luteimonas | unidentified | -7.01 | 0.003 | 0.00 | 0.05 | Environment |
| Pseudomonadota | Gammaproteobacteria | Xanthomonadales | Xanthomonadaceae | Lysobacter | Lysobacter sp. | 22.85 | 0.000 | 0.19 | 0.00 | Environment |
| Verrucomicrobiota | Verrucomicrobiae | Chthoniobacterales | Chthoniobacteraceae | LD29 | uncultured | 2.84 | 0.002 | 0.77 | 0.16 | Environment |
| **Heat treatment (H) Vs. Humidity treatment (Hum)** | | | | | | | |  |  |  |
| **Phylum** | **Class** | **Order** | **Family** | **Genus** | **Species** | **log2FoldChange** | **pvalue** | **H(%)** | **Hum(%)** | **Source** |
| Acidobacteriota | Acidobacteriae | Acidobacteriales | Acidobacteriaceae | Granulicella | uncultured | 19.82 | 0.000 | 0.00 | 0.00 | Environment |
| Actinomycetota | Acidimicrobiia | Microtrichales | Microtrichaceae | IMCC26207 | unidentified | 19.08 | 0.000 | 0.00 | 0.00 | Environment |
| Actinomycetota | Actinobacteria | Actinomycetales | Actinomycetaceae | Flaviflexus | uncultured | -23.35 | 0.000 | 0.00 | 0.07 | Animal |
| Actinomycetota | Actinobacteria | Corynebacteriales | Corynebacteriaceae | Corynebacterium | C. marinum | -24.95 | 0.000 | 0.00 | 0.14 | Environment |
| Actinomycetota | Actinobacteria | Micrococcales | Bogoriellaceae | Georgenia | unidentified | -23.14 | 0.000 | 0.00 | 0.05 | Environment |
| Actinomycetota | Actinobacteria | Micrococcales | Intrasporangiaceae | Ornithinicoccus | uncultured | -37.30 | 0.000 | 0.00 | 0.18 | Environment |
| Actinomycetota | Actinobacteria | Micrococcales | Intrasporangiaceae | Ornithinimicrobium | unidentified | -29.67 | 0.000 | 0.00 | 0.09 | Environment |
| Actinomycetota | Actinobacteria | Micrococcales | Micrococcaceae | Yaniella | uncultured | -26.97 | 0.000 | 0.00 | 0.04 | Environment |
| Actinomycetota | Actinobacteria | Micrococcales | unidentified | unidentified | unidentified | 23.29 | 0.000 | 0.10 | 0.00 | Environment |
| Actinomycetota | Actinobacteria | Propionibacteriales | Nocardioidaceae | Nocardioides | N. gilvus | -25.56 | 0.000 | 0.00 | 0.05 | Environment |
| Bacillota | Bacilli | Bacillales | Planococcaceae | Planococcus | uncultured | -37.07 | 0.000 | 0.00 | 0.25 | Environment |
| Bacillota | Bacilli | Bacillales | Planococcaceae | Psychrobacillus | unidentified | -4.52 | 0.001 | 0.03 | 0.46 | Environment |
| Bacillota | Bacilli | Bacillales | Planococcaceae | unidentified | unidentified | -5.97 | 0.002 | 0.01 | 0.36 | Environment |
| Bacillota | Bacilli | Lactobacillales | Aerococcaceae | Facklamia | unidentified | -24.55 | 0.000 | 0.00 | 0.09 | Animal,pathogen |
| Bacillota | Bacilli | Paenibacillales | Paenibacillaceae | Paenibacillus | unidentified | -5.79 | 0.000 | 0.00 | 0.12 | Environment |
| Bacillota | Bacilli | Staphylococcales | Staphylococcaceae | Aliicoccus | uncultured | -38.00 | 0.000 | 0.00 | 0.12 | Environment |
| Bacillota | Bacilli | Staphylococcales | Staphylococcaceae | Jeotgalicoccus | unidentified | -7.48 | 0.000 | 0.00 | 0.66 | Environment |
| Bacillota | Clostridia | Lachnospirales | Lachnospiraceae | Blautia | B. glucerasea | -21.31 | 0.000 | 0.00 | 0.03 | Animal gut |
| Bacteroidota | Bacteroidia | Flavobacteriales | Flavobacteriaceae | Aequorivita | A. capsosiphonis | 24.63 | 0.000 | 0.22 | 0.00 | Environment |
| Bacteroidota | Bacteroidia | Flavobacteriales | Flavobacteriaceae | Gelidibacter | bacterium_HAB15 | 24.64 | 0.000 | 0.22 | 0.00 | Environment |
| Bacteroidota | Bacteroidia | Flavobacteriales | Flavobacteriaceae | Gelidibacter | uncultured | 16.30 | 0.000 | 0.00 | 0.00 | Environment |
| Chloroflexota | Chloroflexotaa | Thermomicrobiales | JG30-KF-CM45 | JG30-KF-CM45 | bacterium_QTYC46b | -26.09 | 0.000 | 0.00 | 0.03 | Environment |
| Cyanobacteria | Cyanobacteriia | Cyanobacteriales | Nostocaceae | Scytonema_UTEX_2349 | unidentified | -20.28 | 0.000 | 0.00 | 0.06 | Environment |
| Fusobacteriota | Fusobacteriia | Fusobacteriales | Fusobacteriaceae | Fusobacterium | Fusobacterium_sp. | 26.69 | 0.000 | 0.05 | 0.00 | Animal flora, pathogen |
| Gemmatimonadota | Longimicrobia | Longimicrobiales | Longimicrobiaceae | Longimicrobium | unidentified | 21.32 | 0.000 | 0.01 | 0.00 | Environment |
| Pseudomonadota | Alphaproteobacteria | Caulobacterales | Caulobacteraceae | Brevundimonas | B.diminuta | 22.11 | 0.000 | 0.05 | 0.00 | Plant pathogen |
| Pseudomonadota | Alphaproteobacteria | Rhizobiales | Rhizobiaceae | Bartonella | unidentified | -21.47 | 0.000 | 0.00 | 0.04 | Parasite, mite |
| Pseudomonadota | Gammaproteobacteria | Burkholderiales | Alcaligenaceae | Candidimonas | unidentified | 20.97 | 0.000 | 0.01 | 0.00 | Animal,Env. |
| Pseudomonadota | Gammaproteobacteria | Burkholderiales | Alcaligenaceae | Candidimonas | uncultured | 22.25 | 0.000 | 0.04 | 0.00 | Animal,Env. |
| Pseudomonadota | Gammaproteobacteria | Burkholderiales | Comamonadaceae | Simplicispira | unidentified | 23.46 | 0.000 | 0.06 | 0.00 | Environment |
| Pseudomonadota | Gammaproteobacteria | Cardiobacteriales | Wohlfahrtiimonadaceae | Ignatzschineria | Ignatzschineria_sp. | 28.08 | 0.000 | 1.83 | 0.00 | Insects |
| Pseudomonadota | Gammaproteobacteria | Cardiobacteriales | Wohlfahrtiimonadaceae | Ignatzschineria | uncultured | 23.94 | 0.000 | 0.12 | 0.00 | Insects |
| Pseudomonadota | Gammaproteobacteria | Enterobacterales | Morganellaceae | Providencia | P. alcalifaciens | 28.42 | 0.000 | 0.11 | 0.00 | Animal flora |
| Pseudomonadota | Gammaproteobacteria | Enterobacterales | Morganellaceae | Providencia | P. heimbachae | 21.42 | 0.000 | 0.02 | 0.00 | Animal gut |
| Pseudomonadota | Gammaproteobacteria | Enterobacterales | Yersiniaceae | Serratia | S. plymuthica | 27.33 | 0.000 | 0.09 | 0.00 | Env.,Pathogen |
| Pseudomonadota | Gammaproteobacteria | Xanthomonadales | Xanthomonadaceae | Luteimonas | unidentified | -7.83 | 0.001 | 0.00 | 0.05 | Environment |
| Pseudomonadota | Gammaproteobacteria | Xanthomonadales | Xanthomonadaceae | Lysobacter | Lysobacter_sp. | 22.25 | 0.000 | 0.04 | 0.00 | Environment |

**Table S5.** Alpha diversity indexes of fungal communities.

| **Nest** | **Treatment** | **Date** | **Observed** | **Chao1** | **Shannon** | **Simpson** |
| --- | --- | --- | --- | --- | --- | --- |
| 175 | Heat | 13 | 566 | 566 | 4.2718903 | 0.94929162 |
| 175 | Heat | 4 | 491 | 491 | 4.6968683 | 0.97340353 |
| 181 | Humidity | 13 | 484 | 484 | 4.31559969 | 0.95137437 |
| 181 | Humidity | 4 | 441 | 441 | 3.78962478 | 0.91791259 |
| 186 | Heat | 13 | 458 | 458 | 3.96384458 | 0.95203434 |
| 186 | Heat | 4 | 561 | 561 | 4.37809618 | 0.96107166 |
| 188 | Control | 13 | 379 | 379 | 4.19598795 | 0.9566227 |
| 188 | Control | 4 | 367 | 367 | 4.17161261 | 0.95836286 |
| 191 | Control | 13 | 393 | 393 | 3.48127199 | 0.87793507 |
| 191 | Control | 4 | 463 | 463 | 4.08393495 | 0.95111669 |
| 193 | Humidity | 13 | 390 | 390 | 4.39029049 | 0.9674563 |
| 193 | Humidity | 4 | 472 | 472 | 4.30283672 | 0.96626995 |
| 27 | Heat | 13 | 466 | 466 | 4.48765124 | 0.9613526 |
| 27 | Heat | 4 | 461 | 461 | 4.25438965 | 0.96325069 |
| 296 | Control | 13 | 361 | 361 | 2.45174711 | 0.66126857 |
| 296 | Control | 4 | 290 | 290 | 3.47012512 | 0.9223163 |
| 77 | Humidity | 13 | 510 | 510 | 4.00208763 | 0.95053634 |
| 77 | Humidity | 4 | 385 | 385 | 2.7019257 | 0.71214894 |

**Table S6**. Classification of potentially pathogenic bacteria, parasites and symbionts based on FAPROTAX. C: control treatment, Hum: humidity treatment, H: heat treatment.

| **Phylum** | **Taxon** | **Type** | **C (%)** | **Hum (%)** | **H (%)** |
| --- | --- | --- | --- | --- | --- |
| Actinomycetota | *Corynebacterium_minutissimum* | human_patogen, Animal_parasite/gut | 0.01 | 0.00 | 0.00 |
| Actinomycetota | *Cutibacterium_acnes* | human_patogen, animal_parasites_or_symbionts | 0.01 | 0.02 | 0.04 |
| Bacillota | *Clostridium_butyricum* | animal_parasites_or_symbionts | 0.00 | 0.01 | 0.00 |
| Bacillota | *Clostridium_perfringens* | human_patogen animal_parasites_or_symbionts | 0.31 | 0.28 | 0.78 |
| Bacillota | *Enterococcus_faecalis* | human_patogen animal_parasites_or_symbionts | 0.00 | 0.17 | 0.03 |
| Bacillota | *Erysipelatoclostridium* | human_ gastroenteritis | 0.00 | 0.00 | 0.01 |
| Bacillota | *Erysipelothrix_rhusiopathiae* | animal_parasites_or_symbionts | 0.03 | 0.00 | 0.00 |
| Bacillota | *Lactobacillus_reuteri* | animal_parasites_or_symbionts | 0.00 | 0.02 | 0.12 |
| Bacillota | *Ruminococcus_gnavus_group* | animal_parasites_or_symbionts | 0.02 | 0.00 | 0.01 |
| Bacillota | *Ruminococcus_torques_group* | animal_parasites_or_symbionts | 0.04 | 0.01 | 0.02 |
| Bacillota | *Staphylococcus_saprophyticus* | human_patogen, animal_parasites_or_symbionts | 0.01 | 0.01 | 0.00 |
| Bacteroidota | *Empedobacter_brevis* | human_patogen, animal_parasites_or_symbionts | 0.27 | 0.02 | 0.01 |
| Bacteroidota | *Parabacteroides_merdae* | animal_parasites_or_symbionts | 0.01 | 0.00 | 0.00 |
| Campylobacterota | *Providencia_alcalifaciens* | human_ gastroenteritis animal_parasites_or_symbionts | 0.00 | 0.00 | 0.11 |
| Pseudomonadota (Alpha) | *Afipia* | human_patogen animal_parasites_or_symbionts | 0.00 | 0.00 | 0.01 |
| Pseudomonadota (Alpha) | *Roseomonas* | human_patogen animal_parasites_or_symbionts | 0.00 | 0.01 | 0.01 |
| Pseudomonadota (Gamma) | *Acinetobacter_baumannii* | human_pathogens,Septicemia, nosocomia | 0.00 | 0.00 | 0.01 |
| Pseudomonadota (Gamma) | *Acinetobacter_calcoaceticus* | human_nosocomia animal_parasites_or_symbionts | 0.00 | 1.00 | 0.04 |
| Pseudomonadota (Gamma) | *Acinetobacter_lwoffii* | human_pneumonia, nosocomia animal_parasites_or_symbionts | 0.17 | 0.09 | 0.04 |
| Pseudomonadota (Gamma) | *Acinetobacter_radioresistens* | human_nosocomia animal_parasites_or_symbionts | 0.00 | 0.01 | 0.00 |
| Pseudomonadota (Gamma) | *Anaerobiospirillum_succiniciproducens* | animal_parasites_or_symbionts | 0.01 | 0.00 | 0.00 |
| Pseudomonadota (Gamma) | *Burkholderiales_A21b* | human_pathogens pneumonia, animal_parasites_or_symbionts | 0.01 | 0.01 | 0.01 |
| Pseudomonadota (Gamma) | *Burkholderiales_SC-I-84* | human_pneumonia, animal_parasites_or_symbionts | 0.02 | 0.00 | 0.01 |
| Pseudomonadota (Gamma) | *Candidatus_Xiphinematobacter* | animal_parasites_or_symbionts | 0.00 | 0.01 | 0.00 |
| Pseudomonadota (Gamma) | *Candidimonas* | human_pneumonia animal_parasites_or_symbionts | 0.04 | 0.00 | 0.04 |
| Pseudomonadota (Gamma) | *Citrobacter* | animal_parasites_or_symbionts | 0.17 | 0.20 | 0.01 |
| Pseudomonadota (Gamma) | *Duganella* | human_pneumonia animal_parasites_or_symbionts | 0.03 | 0.07 | 0.13 |
| Pseudomonadota (Gamma) | *Enterobacter* | animal_parasites_or_symbionts | 0.00 | 0.01 | 0.02 |
| Pseudomonadota (Gamma) | *Massilia* | human_pneumonia animal_parasites_or_symbionts | 0.00 | 0.01 | 0.01 |
| Pseudomonadota (Gamma) | *Neisseriaceae* | human_pneumonia | 0.01 | 0.00 | 0.05 |
| Pseudomonadota (Gamma) | *Nitrosomonadaceae_966-1* | animal_parasites_or_symbionts | 0.00 | 0.00 | 0.01 |
| Pseudomonadota (Gamma) | *Nitrosomonadaceae_Ellin6067* | human_pneumonia, animal_parasites_or_symbionts | 0.00 | 0.01 | 0.02 |
| Pseudomonadota (Gamma) | *Pandoraea* | human_pneumonia animal_parasites_or_symbionts | 0.00 | 0.00 | 0.01 |
| Pseudomonadota (Gamma) | *Pandoraea* | human_patogen animal_parasites_or_symbionts | 0.00 | 0.00 | 0.01 |
| Pseudomonadota (Gamma) | *Pseudomonas_viridiflava* | plant_pathogen | 0.00 | 0.01 | 0.00 |
| Pseudomonadota (Gamma) | *Sutterella* | animal_parasites_or_symbionts | 0.01 | 0.00 | 0.00 |
| Pseudomonadota (Gamma) | *Verticiella* | human_pneumonia animal_parasites_or_symbionts | 0.00 | 0.02 | 0.03 |
| Pseudomonadota (Gamma) | *Vitreoscilla* | animal_parasites_or_symbionts | 0.01 | 0.00 | 0.05 |
| Pseudomonadota (Gamma) | *Xylophilus* | human_pneumonia animal_parasites_or_symbionts | 0.00 | 0.04 | 0.03 |
| Pseudomonadota (Gamma) | *Yersinia_enterocolitica* | invertebrate_parasites, animal_parasites_or_symbionts | 0.05 | 0.00 | 0.00 |

**Table S8.** Classification of potentially pathogenic animal-associated fungi with higher relative abundance in humidity nests based on FUNGuild analysis. C: control treatment, Hum: humidity treatment, H: heat treatment.

| **Taxon_UNITE** | **Taxon_ FUNGUILD** | **trophicMode** | **guild** | **C (%)** | **Hum (%)** | **H (%)** |
| --- | --- | --- | --- | --- | --- | --- |
| *Lecythophora_canina* | Coniochaetaceae | Pathotroph-Saprotroph-Symbiotroph | Animal Pathogen-Dung Saprotroph-Endophyte-Lichen Parasite-Plant Pathogen-Undefined Saprotroph | 0.009 | 0.022 | 0.004 |
| *Wickerhamomyces_anomalus* | *Wickerhamomyces anomalus* | Pathotroph | Animal Pathogen | 0.000 | 0.005 | 0.002 |
| *Candida_argentea* | *Candida* | Pathotroph-Saprotroph-Symbiotroph | Animal Pathogen-Endophyte-Endosymbiont-Epiphyte-Soil Saprotroph-Undefined Saprotroph | 0.000 | 0.001 | 0.000 |
| *Candida_melibiosica* | *Candida* | Pathotroph-Saprotroph-Symbiotroph | Animal Pathogen-Endophyte-Endosymbiont-Epiphyte-Soil Saprotroph-Undefined Saprotroph | 0.000 | 0.005 | 0.000 |
| *Fusarium_hostae* | Nectriaceae | Pathotroph-Saprotroph-Symbiotroph | Animal Pathogen-Endophyte-Fungal Parasite-Lichen Parasite-Plant Pathogen-Wood Saprotroph | 0.000 | 0.001 | 0.000 |
| *Rhodotorula_dairenensis* | Sporidiobolaceae | Pathotroph-Saprotroph | Animal Pathogen-Undefined Saprotroph | 0.000 | 0.004 | 0.002 |
| *Cladophialophora_nyingchiensis* | Herpotrichiellaceae | Pathotroph-Saprotroph | Animal Pathogen-Fungal Parasite-Undefined Saprotroph | 0.002 | 0.005 | 0.003 |
| *unidentified_Nectriaceae* | Nectriaceae | Pathotroph-Saprotroph-Symbiotroph | Animal Pathogen-Endophyte-Fungal Parasite-Lichen Parasite-Plant Pathogen-Wood Saprotroph | 0.220 | 0.455 | 0.359 |
| *Phialophora_sp* | Herpotrichiellaceae | Pathotroph-Saprotroph | Animal Pathogen-Fungal Parasite-Undefined Saprotroph | 0.000 | 0.002 | 0.000 |
| *Phoma_aloes* | Didymellaceae | Pathotroph-Saprotroph | Animal Pathogen-Plant Pathogen-Undefined Saprotroph | 0.014 | 0.019 | 0.014 |
| *Podonectria_kuwanaspidis* | *Podonectria* | Pathotroph | Animal Pathogen | 0.000 | 0.002 | 0.000 |
| *Coniochaeta_boothii* | Coniochaetaceae | Pathotroph-Saprotroph-Symbiotroph | Animal Pathogen-Dung Saprotroph-Endophyte-Lichen Parasite-Plant Pathogen-Undefined Saprotroph | 0.000 | 0.002 | 0.000 |
| *Dactylonectria_macrodidyma* | Nectriaceae | Pathotroph-Saprotroph-Symbiotroph | Animal Pathogen-Endophyte-Fungal Parasite-Lichen Parasite-Plant Pathogen-Wood Saprotroph | 0.000 | 0.005 | 0.000 |
| *unidentified_Didymellaceae* | Didymellaceae | Pathotroph-Saprotroph | Animal Pathogen-Plant Pathogen-Undefined Saprotroph | 0.719 | 1.762 | 1.518 |
| *Nigrograna_obliqua* | *Nigrograna* | Pathotroph | Animal Pathogen | 0.000 | 0.005 | 0.000 |
| *Simplicillium_lamellicola* | *Simplicillium* | Pathotroph | Animal Pathogen | 0.000 | 0.017 | 0.001 |
| *Meristemomyces_sp* | Teratosphaeriaceae | Pathotroph-Saprotroph | Animal Pathogen-Plant Pathogen-Undefined Saprotroph | 0.007 | 0.033 | 0.007 |
| *Trichosporon_lactis* | *Trichosporon* | Pathotroph | Animal Pathogen | 0.000 | 0.117 | 0.108 |
| *Candida_palmioleophila* | *Candida* | Pathotroph-Saprotroph-Symbiotroph | Animal Pathogen-Endophyte-Endosymbiont-Epiphyte-Soil Saprotroph-Undefined Saprotroph | 0.000 | 0.006 | 0.000 |
| *Fusicolla_septimanifiniscientiae* | Nectriaceae | Pathotroph-Saprotroph-Symbiotroph | Animal Pathogen-Endophyte-Fungal Parasite-Lichen Parasite-Plant Pathogen-Wood Saprotroph | 0.001 | 0.118 | 0.012 |
| *Haptocillium_sinense* | *Haptocillium* | Pathotroph | Animal Pathogen | 0.000 | 0.003 | 0.002 |
| *Aspergillus_penicillioides* | *Aspergillus penicillioides* | Pathotroph | Animal Pathogen | 0.001 | 0.017 | 0.002 |
| *Constantinomyces_oldenburgensis* | Teratosphaeriaceae | Pathotroph-Saprotroph | Animal Pathogen-Plant Pathogen-Undefined Saprotroph | 0.008 | 0.040 | 0.011 |
| *Gibberella_baccata* | Nectriaceae | Pathotroph-Saprotroph-Symbiotroph | Animal Pathogen-Endophyte-Fungal Parasite-Lichen Parasite-Plant Pathogen-Wood Saprotroph | 0.000 | 0.213 | 0.040 |
| *Acremonium_charticola* | *Acremonium charticola* | Pathotroph | Animal Pathogen | 0.000 | 0.002 | 0.000 |
| *Didymella_subrosea* | Didymellaceae | Pathotroph-Saprotroph | Animal Pathogen-Plant Pathogen-Undefined Saprotroph | 0.000 | 0.007 | 0.000 |
| *Nigrograna_mycophila* | *Nigrograna* | Pathotroph | Animal Pathogen | 0.000 | 0.002 | 0.000 |
| *Neosorocybe_pini* | Herpotrichiellaceae | Pathotroph-Saprotroph | Animal Pathogen-Fungal Parasite-Undefined Saprotroph | 0.000 | 0.003 | 0.000 |
| *Cladophialophora_sylvestris* | Herpotrichiellaceae | Pathotroph-Saprotroph | Animal Pathogen-Fungal Parasite-Undefined Saprotroph | 0.003 | 0.005 | 0.000 |
| *unidentified_Cyphellophora* | Cyphellophoraceae | Pathotroph-Saprotroph | Animal Pathogen-Undefined Saprotroph | 0.000 | 0.003 | 0.000 |
| *unidentified_Volutella* | Nectriaceae | Pathotroph-Saprotroph-Symbiotroph | Animal Pathogen-Endophyte-Fungal Parasite-Lichen Parasite-Plant Pathogen-Wood Saprotroph | 0.003 | 0.015 | 0.000 |
| *Cyphellophora_sp* | Cyphellophoraceae | Pathotroph-Saprotroph | Animal Pathogen-Undefined Saprotroph | 0.000 | 0.055 | 0.043 |
| *Pseudotaeniolina_globosa* | Teratosphaeriaceae | Pathotroph-Saprotroph | Animal Pathogen-Plant Pathogen-Undefined Saprotroph | 0.001 | 0.001 | 0.001 |
| *Cyphellophoraceae_sp* | Cyphellophoraceae | Pathotroph-Saprotroph | Animal Pathogen-Undefined Saprotroph | 0.000 | 0.006 | 0.005 |
| *Acremonium_camptosporum* | *Acremonium* | Pathotroph-Saprotroph-Symbiotroph | Animal Pathogen-Endophyte-Fungal Parasite-Plant Pathogen-Wood Saprotroph | 0.000 | 0.001 | 0.000 |
| *unidentified_Tubeufiaceae* | Tubeufiaceae | Pathotroph-Saprotroph | Animal Pathogen-Undefined Saprotroph | 0.056 | 0.109 | 0.118 |
| *Neonectria_major* | Nectriaceae | Pathotroph-Saprotroph-Symbiotroph | Animal Pathogen-Endophyte-Fungal Parasite-Lichen Parasite-Plant Pathogen-Wood Saprotroph | 0.001 | 0.023 | 0.005 |
| *Coniochaeta_cipronana* | Coniochaetaceae | Pathotroph-Saprotroph-Symbiotroph | Animal Pathogen-Dung Saprotroph-Endophyte-Lichen Parasite-Plant Pathogen-Undefined Saprotroph | 0.027 | 0.059 | 0.018 |
| *unidentified_Exophiala* | Herpotrichiellaceae | Pathotroph-Saprotroph | Animal Pathogen-Fungal Parasite-Undefined Saprotroph | 0.019 | 0.020 | 0.010 |
| *Nectria_ramulariae* | Nectriaceae | Pathotroph-Saprotroph-Symbiotroph | Animal Pathogen-Endophyte-Fungal Parasite-Lichen Parasite-Plant Pathogen-Wood Saprotroph | 0.000 | 1.139 | 0.006 |
| *Sporobolomyces_sp* | Sporidiobolaceae | Pathotroph-Saprotroph | Animal Pathogen-Undefined Saprotroph | 0.000 | 0.003 | 0.001 |
| *Lachancea_thermotolerans* | *Lachancea* | Pathotroph | Animal Pathogen | 0.004 | 0.009 | 0.001 |
| *Exophiala_xenobiotica* | Herpotrichiellaceae | Pathotroph-Saprotroph | Animal Pathogen-Fungal Parasite-Undefined Saprotroph | 0.000 | 0.001 | 0.000 |
| *Veronaea_sp* | Herpotrichiellaceae | Pathotroph-Saprotroph | Animal Pathogen-Fungal Parasite-Undefined Saprotroph | 0.001 | 0.003 | 0.002 |
| *Myriangium_sp* | *Myriangium* | Pathotroph | Animal Pathogen | 0.000 | 0.010 | 0.000 |
| *Coniochaeta_elegans* | Coniochaetaceae | Pathotroph-Saprotroph-Symbiotroph | Animal Pathogen-Dung Saprotroph-Endophyte-Lichen Parasite-Plant Pathogen-Undefined Saprotroph | 0.000 | 0.047 | 0.000 |
| *Neoascochyta_triticicola* | Didymellaceae | Pathotroph-Saprotroph | Animal Pathogen-Plant Pathogen-Undefined Saprotroph | 0.017 | 0.142 | 0.030 |
| *Neocladophialophora_quercina* | Herpotrichiellaceae | Pathotroph-Saprotroph | Animal Pathogen-Fungal Parasite-Undefined Saprotroph | 0.000 | 0.001 | 0.000 |
| *Mucor_circinelloides* | *Mucor circinelloides* | Pathotroph | Animal Pathogen-Plant Pathogen | 0.000 | 0.003 | 0.001 |
| *Capnobotryella_sp* | Teratosphaeriaceae | Pathotroph-Saprotroph | Animal Pathogen-Plant Pathogen-Undefined Saprotroph | 0.000 | 0.029 | 0.002 |
| *Fusicolla_sp* | Nectriaceae | Pathotroph-Saprotroph-Symbiotroph | Animal Pathogen-Endophyte-Fungal Parasite-Lichen Parasite-Plant Pathogen-Wood Saprotroph | 0.000 | 0.003 | 0.000 |
| *Aspergillus_ruber* | *Aspergillus ruber* | Pathotroph | Animal Pathogen | 0.009 | 0.011 | 0.002 |
| *Constantinomyces_sp* | Teratosphaeriaceae | Pathotroph-Saprotroph | Animal Pathogen-Plant Pathogen-Undefined Saprotroph | 0.011 | 0.049 | 0.032 |
| *unidentified_Thyronectria* | Nectriaceae | Pathotroph-Saprotroph-Symbiotroph | Animal Pathogen-Endophyte-Fungal Parasite-Lichen Parasite-Plant Pathogen-Wood Saprotroph | 0.000 | 0.013 | 0.000 |
| *Harposporium_janus* | *Harposporium* | Pathotroph | Animal Pathogen | 0.000 | 0.002 | 0.000 |
| *Hirsutella_lecaniicola* | *Hirsutella* | Pathotroph | Animal Pathogen | 0.000 | 0.003 | 0.000 |
| *Cephalotrichum_stemonitis* | Microascaceae | Pathotroph-Saprotroph-Symbiotroph | Animal Pathogen-Endophyte-Plant Pathogen-Undefined Saprotroph | 0.000 | 0.004 | 0.000 |
| *Arthrodermataceae_sp* | Arthrodermataceae | Pathotroph-Saprotroph | Animal Pathogen-Undefined Saprotroph | 0.021 | 0.280 | 0.026 |

**Figure S1**. Relative abundances of bacterial sequences at genus level (only those with relative abundances greater than 1% are represented). Nest identification number and the moment of sampling (at day 4 or day 13 of nestling age) corresponding to each sample is shown at the bottom of the figure. The numbers represent the relative abundance of each genus per sample.


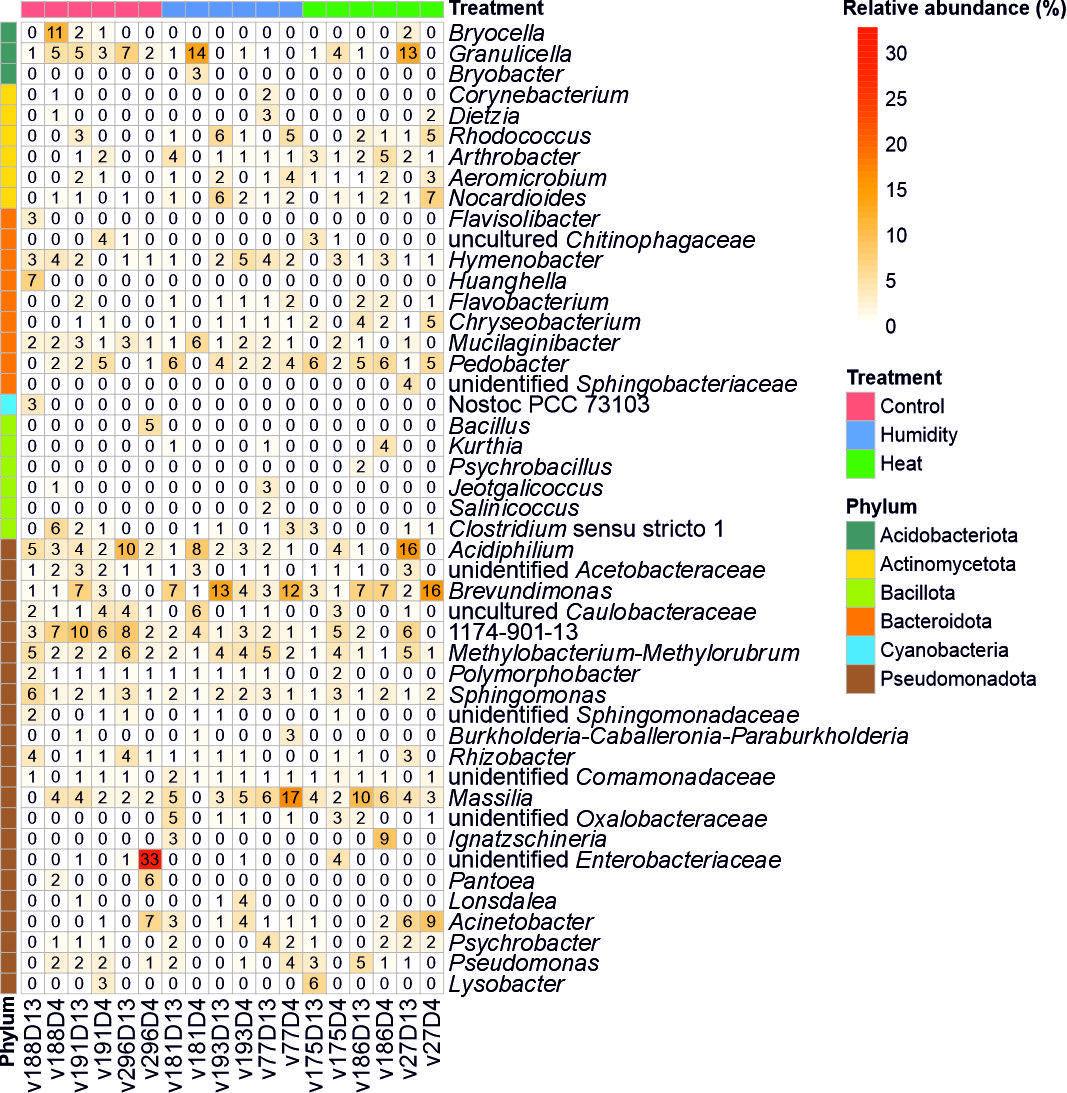


**Figure S2**. Relative abundance of bacteria with relative abundances greater than 15% grouped by their main ecological functions and by treatment based on FAPROTAX. The boxplot represents medians, quartiles (Q1 and Q3), whiskers showing the maximum and minimum values, and outliers.
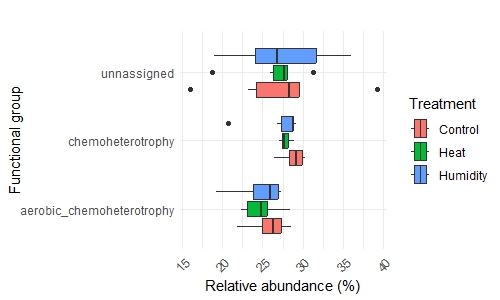


**Figure S3**. Relative abundance of bacteria with relative abundances between 1% and 15% grouped by their main ecological functions and by treatment based on FAPROTAX. The boxplot represents medians, quartiles (Q1 and Q3), whiskers showing the maximum and minimum values, and outliers.
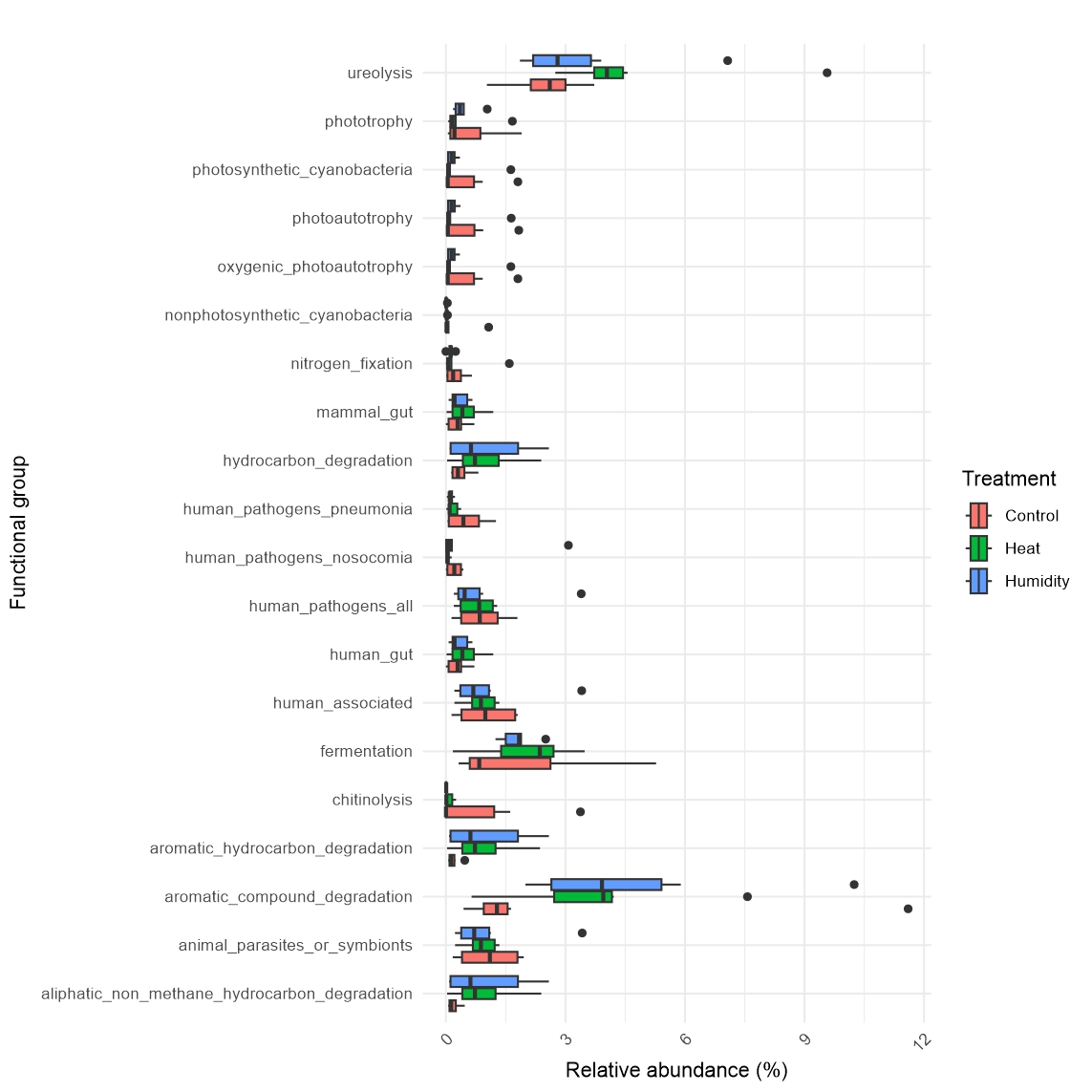


**Figure S4**. Relative abundances of fungal sequences at species level (only those with relative abundances greater than 2% are represented). Nest identification number and the moment of sampling (at day 4 or day 13 of nestling age) corresponding to each sample is shown at the bottom of the figure. The numbers represent the relative abundance of each genus per sample.


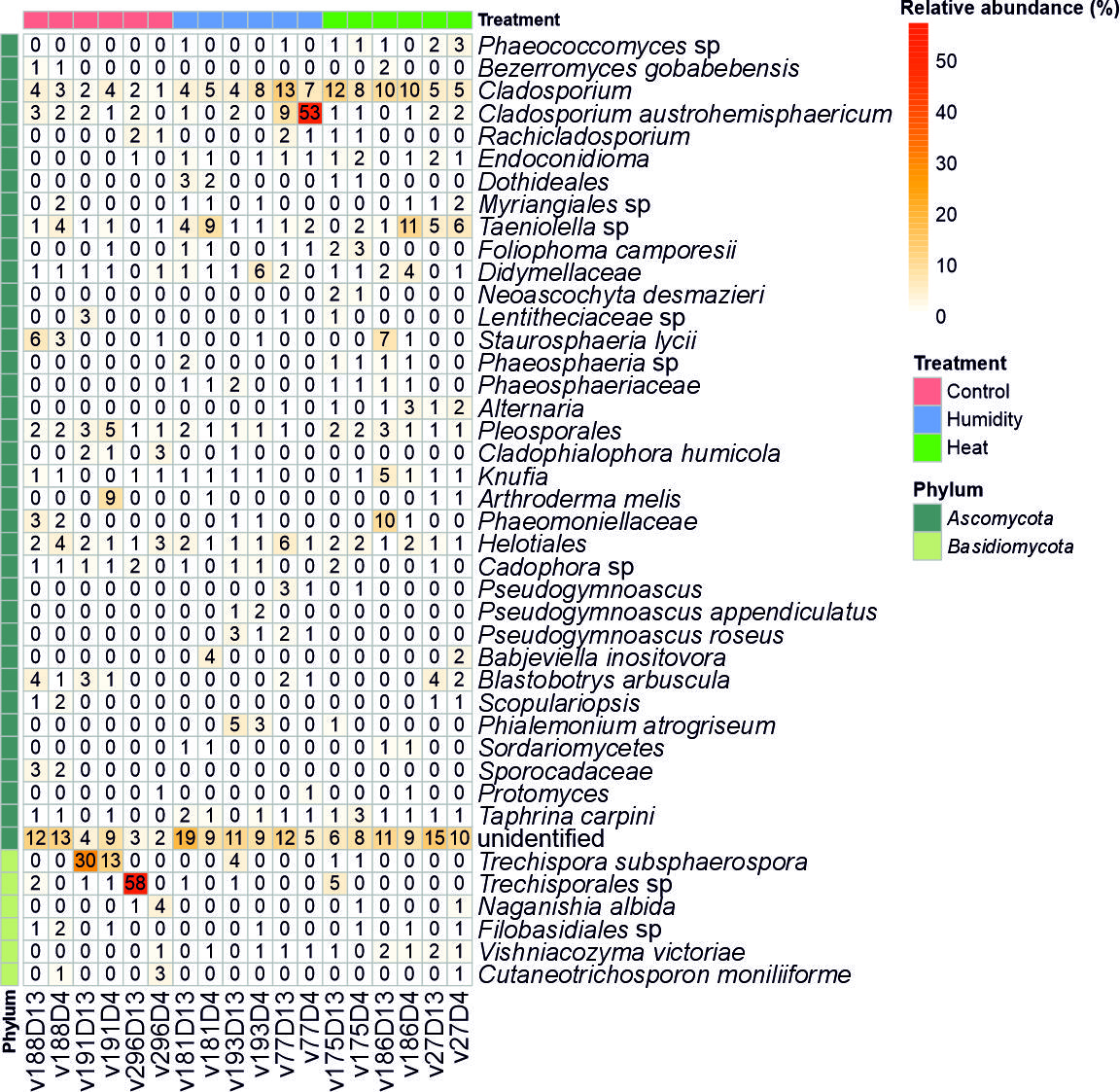


**Figure S5**. Relative abundance of fungi grouped by their main ecological functions and by treatment based on FUNGuild. The boxplot represents medians, quartiles (Q1 and Q3), whiskers showing the maximum and minimum values, and outliers.


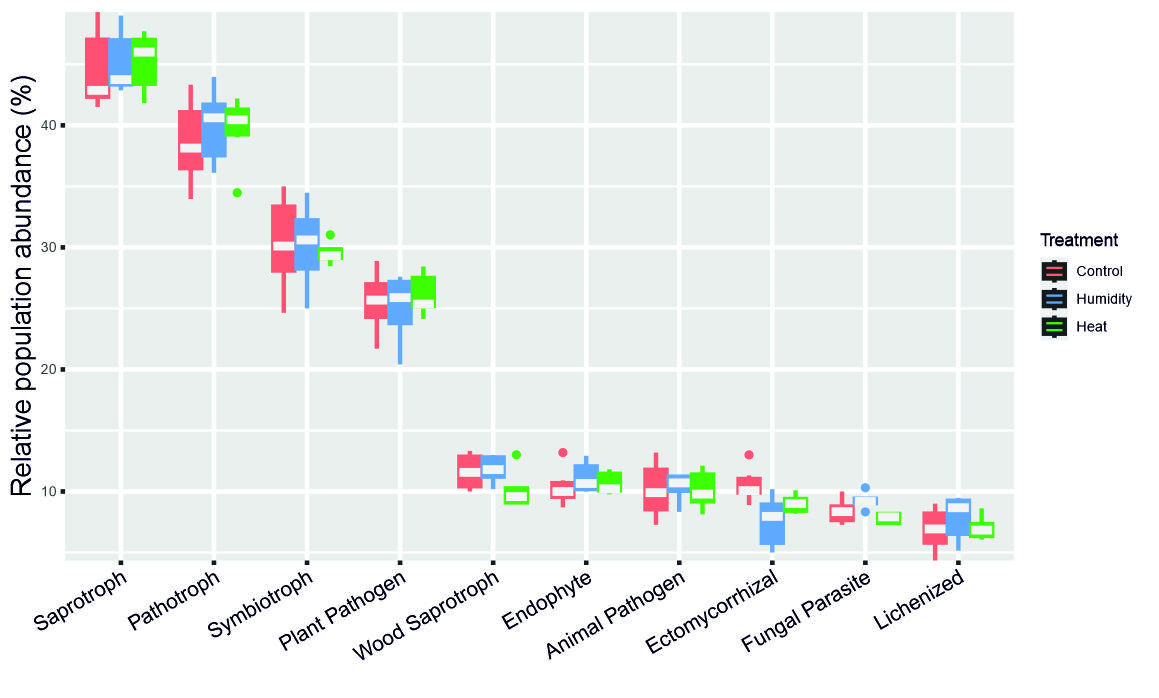

Supplement: Supplementary file 1 — Table S1: Results of extraction (Bex) and PCR blanks (Bpcr). Table S2: Alpha diversity indexes of bacterial communities. Table S4: Results of the DESeq analysis for the bacterial communities, grouped by treatment. Table S5: Alpha diversity indexes of fungal communities. Table S6: Classification of potentially pathogenic bacteria, parasites and symbionts based on FAPROTAX. Table S8: Classification of potentially pathogenic animal‐associated fungi with higher relative abundance in humidified nests based on FUNGuild analysis. Figure S1: Relative abundances of bacterial sequences at genus level (only those with relative abundances > 1% are represented). Figure S2: Relative abundance of bacteria with relative abundances > 15% grouped by their main ecological functions and by treatment based on FAPROTAX. Figure S3: Relative abundance of bacteria with relative abundances between 1% and 15% grouped by their main ecological functions and by treatment based on FAPROTAX. Figure S4: Relative abundances of fungal sequences at species level (only those with relative abundances > 2% are represented). Figure S5: Relative abundance of fungi grouped by their main ecological functions and by treatment based on FUNGuild. [file ECE3-16-e74007-s002.docx]
